# Redefining standards: a comprehensive systematic review of practice changing advances in GU oncology from ASCO and ESMO 2025

**DOI:** 10.3389/fendo.2026.1780259

**Published:** 2026-04-01

**Authors:** Nabil Ismaili

**Affiliations:** 1 Department of Medical Oncology, Mohammed VI Faculty of Medicine, Mohammed VI University of Sciences and Health (UM6SS), Mohammed VI Foundation of Sciences and Health (FM6SS), Casablanca, Morocco; 2 Oncopathology, Biology and Environment of Cancer Laboratory, Mohammed VI Center for Research and Innovation (CM6RI), Mohammed VI Foundation of Sciences and Health (FM6SS), Rabat, Morocco

**Keywords:** antibody-drug conjugate, biomarkers, bladder cancer, genitourinary oncology, immunotherapy, kidney cancer, PARP inhibitor, prostate cancer

## Abstract

**Background:**

The year 2025 has been a transformative moment in GU oncology, with paradigm-shifting clinical trial results presented at major conferences and rapidly published. These developments are recasting therapeutic standards across bladder, kidney, prostate, penile, and testicular cancers through novel mechanisms and refined personalization.

**Objectives:**

This systematic review aimed to identify, synthesize, and critically evaluate pivotal phase II and III randomized controlled trials presented at ASCO/ESMO 2025 or published in 2025, focusing on innovative therapeutic strategies across GU malignancies.

**Methods:**

Conducted per PRISMA 2020 guidelines, the review involved exhaustive searches of conference proceedings, PubMed/MEDLINE, and Embase (January-December 2025). Included studies were phase II and III RCTs in GU oncology reporting overall or progression-free survival for novel therapies. Study selection, data extraction, and risk-of-bias assessment using the Cochrane RoB 2 tool were performed.

**Results:**

Forty studies met inclusion criteria: bladder cancer (13), kidney cancer (9), prostate cancer (16), testicular cancer (1), and penile cancer (1). Key advances include: (1) In bladder cancer, perioperative durvalumab (NIAGARA) and enfortumab vedotin plus pembrolizumab (KEYNOTE-905/EV-303) set new standards, while HER2-targeted disitamab vedotin plus toripalimab (RC48-C016) improved metastatic survival. Upfront MRI staging (BladderPath) and kidney-sparing approaches (DISTINCT-I) advanced. (2) In kidney cancer, adjuvant durvalumab (RAMPART) and transcriptomic-guided therapy (OPTIC RCC) were established. LenCabo compared post-immunotherapy regimens, and FRUSICA-2 introduced a novel VEGF-TKI/IO combination. (3) In prostate cancer, enzalutamide plus leuprolide improved survival in high-risk biochemical recurrence (EMBARK). Capivasertib plus abiraterone benefited PTEN-deficient metastatic hormone-sensitive disease (CAPItello-281). The PSMAddition trial demonstrated that adding [^177^Lu]Lu-PSMA-617 to standard therapy significantly improved radiographic PFS in PSMA-positive mHSPC. Docetaxel scheduling was optimized (ARASAFE), and an AI model (STAMPEDE) identified patients for AR inhibitor benefit. Novel agents like saruparib and pasritamig showed promise. (4) In testicular cancer, de-escalated therapy was established for seminoma (SAKK 01/10). (5) In penile cancer, chemotherapy optimization progressed.

**Conclusion:**

The 2025 evidence establishes multiple new standards of care across GU cancers, emphasizing biomarker-driven strategies, immunotherapy integration, novel resistance mechanisms, and treatment optimization. This synthesis provides an evidence-based framework for updating guidelines and highlights the move toward more personalized management, while noting persistent challenges and future research needs.

## 1 Introduction

Genitourinary cancers, encompassing malignancies of the prostate, bladder, kidney, testis, and penis, represent a significant global health burden, accounting for approximately 24% of all cancer diagnoses in men and 8% in women worldwide. The therapeutic landscape for these heterogeneous diseases has historically evolved incrementally, but recent years have witnessed an accelerating pace of discovery driven by advances in molecular biology, tumor immunology, genomics, and targeted drug development.

The year 2025, in particular, has proven to be an exceptionally consequential period, characterized by the presentation of groundbreaking clinical trial results at premier international oncology conferences, most notably the American Society of Clinical Oncology (ASCO) Annual Meeting and the European Society for Medical Oncology (ESMO) Congress, and their concurrent or subsequent publication in high-impact, peer-reviewed medical journals.

This convergence of data dissemination platforms has created a unique and powerful evidence base that is rapidly reconfiguring clinical frameworks across the spectrum of GU malignancies. From the integration of immunotherapy into curative-intent settings for bladder cancer to the breakthrough of novel antibody-drug conjugates (ADCs) in metastatic urothelial carcinoma (mUC) and the application of artificial intelligence to guide therapy in prostate cancer, 2025 has delivered transformative results. Simultaneously, the refinement of biomarker-directed strategies in kidney cancer and the exploration of novel therapeutic mechanisms, such as PARP1-selective inhibitors and bispecific T-cell engagers, have expanded the therapeutic arsenal. Furthermore, important strides in treatment sequencing and optimization underscore a parallel evolution toward reducing treatment-related morbidity without compromising oncologic outcomes.

Despite this wealth of new data, the rapid and simultaneous presentation of these studies across multiple venues presents a challenge for clinicians, researchers, and policymakers seeking to synthesize and critically appraise this evidence to inform practice and research directions. There exists a clear need for a comprehensive, systematic evaluation that integrates conference-derived data with peer-reviewed publications, assesses methodological quality, and contextualizes findings within the evolving therapeutic landscape.

This systematic review, therefore, aims to provide a rigorous synthesis and critical appraisal of the most significant therapeutic advances in GU oncology reported throughout 2025. We focus specifically on phase II and III randomized controlled trials (RCTs) that have the highest potential to redefine clinical standards of care. By systematically integrating data from both major conference presentations and their rapid full-length publications, this review offers a timely, consolidated, and evidence-based overview of the current state of the art. It is structured to guide stakeholders by detailing novel therapeutic strategies, elucidating the increasingly central role of biomarkers in clinical decision-making, discussing implications for personalized patient care across different disease sites and stages, and identifying key knowledge gaps and future research priorities. Ultimately, this synthesis seeks to translate a year of remarkable scientific progress into a coherent framework for advancing patient care in GU oncology.

## 2 Methods

### 2.1 Protocol and registration

This systematic review was designed and conducted in strict accordance with the Preferred Reporting Items for Systematic Reviews and Meta-Analyses (PRISMA) 2020 statement to ensure methodological rigor, transparency, and reproducibility. A detailed study protocol outlining the research question, search strategy, eligibility criteria, data extraction methodology, and planned synthesis approach was developed *a priori*. In accordance with PRISMA guidelines for rapid reviews of high-impact conference data, this protocol was not registered on PROSPERO but was strictly followed.

### 2.2 Eligibility criteria

Studies were included or excluded based on the following predefined criteria, designed to capture high-level evidence of novel therapeutic interventions across GU malignancies.

#### 2.2.1 Inclusion criteria

Studies were considered for inclusion if they were Phase II or III RCTs. This focus was chosen to prioritize evidence from controlled studies with comparative efficacy data, which form the basis for regulatory approvals and practice-changing recommendations. The population of interest was adult patients (aged 18 years or older) with a histologically confirmed diagnosis of a primary GU malignancy, including bladder, kidney, prostate, testicular, or penile cancer. Studies of mixed populations were included only if results were reported separately for the relevant GU cancer cohort.

The intervention and comparison had to involve the evaluation of a novel therapeutic agent, combination regimen, or treatment strategy (experimental arm) compared to a contemporary standard of care, an active comparator, or placebo (control arm). “Novel” was defined as involving a drug or strategy not yet established in the specific clinical setting under investigation by international guidelines at the start of 2025. Furthermore, included studies were required to report at least one primary efficacy endpoint of overall survival (OS) or progression-free survival (PFS), as assessed by investigator or blinded independent central review (BICR). Overall survival, defined as the time from randomization to death from any cause, was considered the gold-standard efficacy endpoint. Progression-free survival, defined as time from randomization to disease progression or death from any cause, was accepted as a clinically relevant primary endpoint, particularly in maintenance or frontline therapy trials.

The source of evidence was a key criterion. To capture the most current and impactful data, study results were eligible only if they were presented as an oral or late-breaking presentation at the ASCO 2025 Annual Meeting or the ESMO Congress 2025. Furthermore, rapid peer-reviewed publications of these specific conference presentations with a 2025 publication date were also eligible. All studies needed to be in English.

#### 2.2.2 Exclusion criteria

Studies were excluded based on several factors. In general, Phase I trials and non-randomized study designs (including single-arm phase II trials) were excluded due to their focus on safety/dose-finding or lack of comparative efficacy data. However, exceptions were made on a case-by-case basis for a limited number of phase I/II studies if they provided first-in-class proof-of-concept for a novel mechanism or therapeutic strategy judged to be of high clinical importance, even in the absence of a randomized control. We excluded studies that did not report hazard ratios (HRs) with confidence intervals (CIs) or sufficient data to calculate them for OS or PFS. Duplicate publications or multiple reports from the same trial cohort were identified, and in such cases, the most comprehensive, recent, or final report was selected. We also excluded preclinical studies, *in vitro* investigations, retrospective observational studies, cost-effectiveness analyses, qualitative studies, and reviews. Lastly, studies focused exclusively on supportive care, imaging techniques, surgical techniques without oncologic outcomes, or diagnostic biomarkers not linked to a therapeutic intervention were not included.

### 2.3 Information sources and search strategy

A comprehensive, multi-source search strategy was employed between December 1 and December 20, 2025, to minimize the risk of missing relevant studies.

#### 2.3.1 Conference proceedings

The official online abstract libraries and virtual meeting platforms for the ASCO 2025 Annual Meeting and the ESMO Congress 2025 were systematically searched. All abstract categories, including Late-Breaking Abstracts, were reviewed to capture all relevant presentations from these premier oncology conferences.

#### 2.3.2 Electronic databases

The search strategy was designed to prioritize data presented at the premier annual oncology conferences. We first conducted a systematic review of all oral and late-breaking presentations within the Genitourinary Oncology tracks of the ASCO 2025 Annual Meeting and the ESMO Congress 2025 official programs and abstract libraries.

Subsequently, to identify peer-reviewed rapid publications stemming from these conference presentations, targeted searches were performed in PubMed/MEDLINE and Embase from January 1 to December 20, 2025. Search strings were developed with a medical librarian and tailored to each database. They combined Medical Subject Headings (MeSH) and free-text keywords for key concepts: (1) GU cancer types, (2) clinical trial designs (e.g., “Randomized Controlled Trial”), and (3) the 2025 conferences themselves (e.g., “ASCO 2025”). This approach was designed to systematically locate journal articles corresponding to the conference abstracts. Boolean operators (AND, OR) were used to combine these terms effectively.

#### 2.3.3 Hand-searching and complementary searches

To ensure the comprehensive capture of rapid publications stemming from the ASCO and ESMO 2025 meetings, we performed complementary manual searches. First, we hand-searched the tables of contents of major general medical and oncology journals (e.g., New England Journal of Medicine, The Lancet, Journal of Clinical Oncology, The Lancet Oncology, Annals of Oncology) for issues published in 2025 to identify any full-text articles corresponding to the conference abstracts of interest. Second, we scrutinized clinical trial registries (ClinicalTrials.gov, EU Clinical Trials Register) for results postings or updates in 2025 related to trials presented at these conferences. Finally, we screened the reference lists of all included studies and relevant review articles to identify any additional eligible records that our primary search might have missed.

### 2.4 Study selection process

The study selection process was conducted in a structured, two-stage manner to ensure methodological rigor and minimize bias. All records retrieved from the searches were imported into Covidence systematic review software, where duplicates were automatically and manually removed.

#### 2.4.1 Title and abstract screening

The author screened the titles and abstracts of all unique records against the eligibility criteria. To validate these initial decisions, a second, independent verification of the screening (inclusion/exclusion) for all records was subsequently performed. Records that clearly did not meet the criteria (e.g., preclinical study, wrong cancer type) were excluded. All records deemed potentially relevant or where eligibility was uncertain based on the abstract proceeded to full-text review.

#### 2.4.2 Full-text review

**Figure 1:**
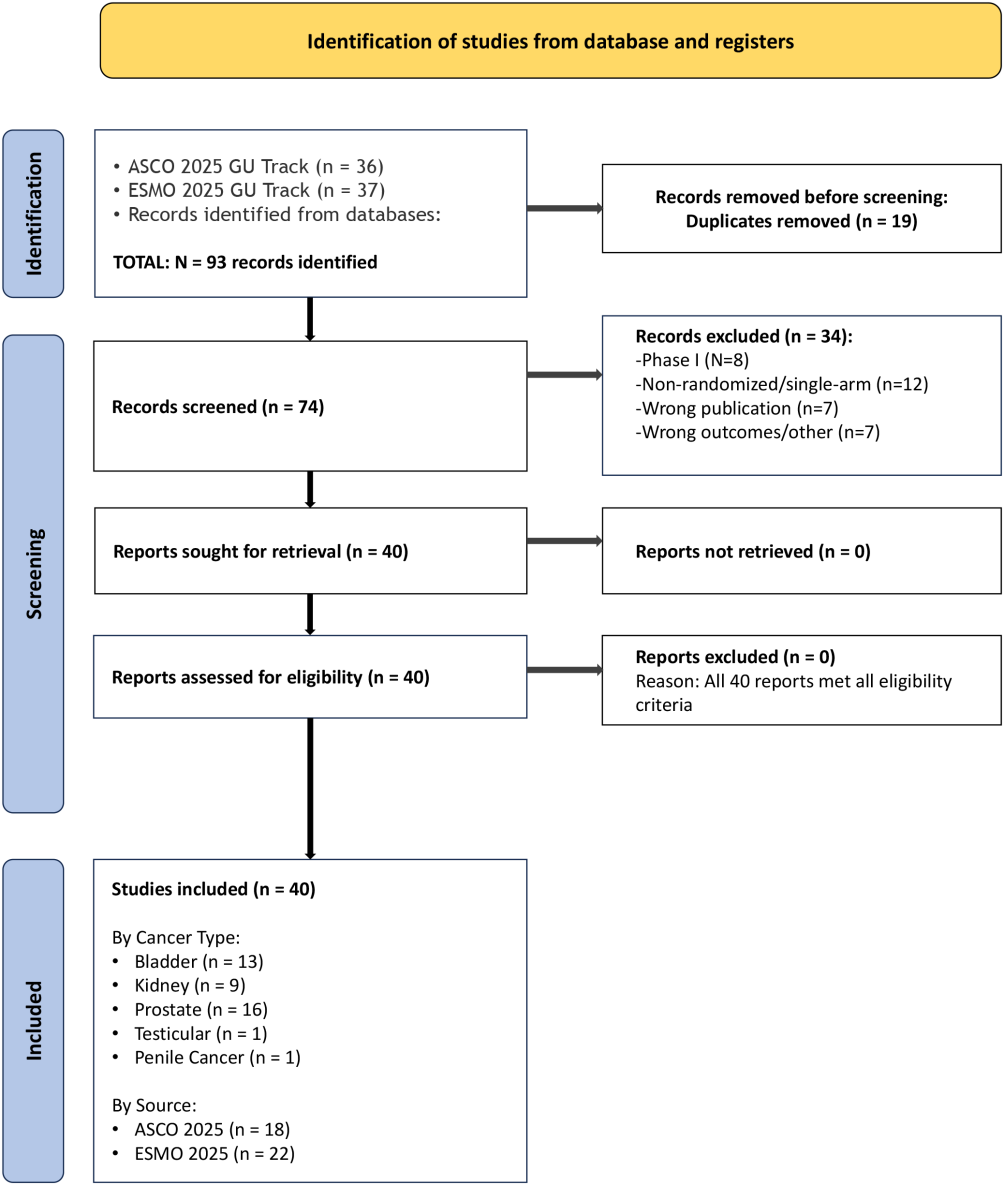
PRISMA 2020 flow diagram of study selection for systematic review of genitourinary oncology advances in 2025. This figure visually summarizes the systematic process of identifying, screening, and selecting studies for this review. Comprehensive searches were conducted across the Genitourinary Oncology tracks of the ASCO 2025 Annual Meeting (n=36) and ESMO 2025 Congress (n=37), supplemented by database searches. After removing 19 duplicate records, 74 unique records underwent title and abstract screening. Following full-text assessment of 40 reports, all 40 studies met the pre-defined eligibility criteria and were included in the final qualitative synthesis. The distribution across tumor types was: Bladder Cancer (n=13), Kidney Cancer (n=9), Prostate Cancer (n=16), Testicular Cancer (n=1), and Penile Cancer (n=1), for a total of 40 studies. This balanced representation reflects the major therapeutic advances presented at both premier oncology conferences in 2025.

The full-text articles, conference presentation slides, and/or detailed abstract supplements for all records passing the initial screen were retrieved. The author assessed these documents against the full eligibility criteria. This assessment was then subjected to an independent verification. Reasons for exclusion at this stage were systematically documented for each record (see PRISMA flow diagram, **Figure 1**). Any discrepancies identified during the verification process were resolved through careful re-evaluation of the study against the predefined criteria until a final, consistent decision was reached.

During the full-text review, a dedicated search was performed in PubMed/MEDLINE and Embase for each study initially identified as an ASCO 2025 or ESMO 2025 conference abstract. This step aimed to identify any corresponding full-length, peer-reviewed publication with a 2025 date, thereby capturing the most mature data. Ultimately, 9 of the 40 included studies (22.5%) were available as full publications by our search cutoff (December 20, 2025), and are cited as such. It is critical to emphasize that all studies included in this synthesis were first presented at either the ASCO or ESMO 2025 meetings, ensuring this review captures the most current evidence from these premier conferences.

### 2.5 Data extraction and management

Data from included studies were extracted by the author using a standardized, piloted electronic data extraction form. A subsequent independent verification of all extracted data was performed to ensure accuracy.

The extracted data encompassed several key domains. Study characteristics included the trial name or identifier, ClinicalTrials.gov registration number, first author or presenter, year of presentation or publication, journal or conference name, study phase, design, primary endpoint(s), statistical design, and duration of follow-up. Participant characteristics covered the total sample size, number of patients per arm, median age, disease stage or setting, histology, number and type of prior lines of therapy, and key biomarker statuses. These biomarkers included PD-L1 expression, mismatch repair and microsatellite instability status, homologous recombination deficiency status, BRCA mutation status, PTEN status, HER2 expression, and other specific genetic alterations. Intervention details involved a description of the experimental and control regimens, including drug names, doses, schedules, route of administration, treatment duration, and permitted supportive care. Outcome data focused on primary results such as hazard ratios with confidence intervals and p-values for overall and progression-free survival, median survival times, as well as secondary efficacy and safety outcomes. The latter included objective response rate (ORR), duration of response, pathologic complete (pCRs) rates, and adverse event (AE) profiles. Other relevant data captured key stratification factors, prespecified subgroup analyses, statistical analysis plan details, source of funding, and declared conflicts of interest.

### 2.6 Risk of bias assessment

The methodological quality and risk of bias for each included randomized controlled trial were assessed by the author using the revised Cochrane Risk of Bias tool for randomized trials (RoB 2). To ensure consistency and minimize subjective bias, all assessments underwent a structured, independent verification process.

This tool evaluates five key domains: bias arising from the randomization process; bias due to deviations from the intended interventions; bias due to missing outcome data; bias in the measurement of the outcome; and bias in the selection of the reported result. For each domain, a judgment of “Low risk,” “Some concerns,” or “High risk” of bias was made based on a series of signaling questions, and an overall risk-of-bias judgment was then derived for each study.

For studies initially reported as conference abstracts, the assessment was based on the methodological details provided in the abstract and any accompanying presentation slides or supplemental materials. Where these details were insufficient to judge a domain definitively, it was conservatively rated as having “Some concerns.” Any uncertainties arising during the assessment or verification process were meticulously re-examined in light of the protocol and tool guidelines until a final, consistent judgment was reached. The results of this risk of bias assessment are summarized in both tabular and graphical form in the results section.

### 2.7 Data synthesis

Given the anticipated clinical, methodological, and statistical heterogeneity across the included studies, stemming from differences in cancer types, disease stages, prior treatments, therapeutic mechanisms, comparator arms, and biomarker definitions, a formal quantitative meta-analysis was deemed neither appropriate nor feasible. The primary objective was instead to provide a comprehensive narrative synthesis of the evidence.

A structured narrative synthesis approach was therefore adopted. This process began with a description of studies, including a tabular and narrative summary of their characteristics, populations, interventions, and outcomes. The findings were then organized thematically, first by primary disease site in the order of bladder cancer, kidney cancer, prostate cancer, and penile cancer. Within each disease site, studies were further grouped by clinical setting, such as localized/curative-intent disease, perioperative management, metastatic first-line therapy, maintenance therapy, and later-line treatments.

For each major trial or group of trials, the synthesis describes the rationale and study design; key efficacy results with a focus on hazard ratios, median survival times, and statistical significance; important subgroup analyses based on biomarkers; key safety findings; and the authors’ main conclusions. The critical appraisal, incorporating the risk of bias assessment and limitations of the evidence like the interim nature of data or open-label design, is integrated into the narrative for each major finding. Furthermore, the synthesis aims to identify overarching themes, concordances, and discordances across studies, particularly regarding biomarker utility, sequencing of therapies, and emerging mechanisms of resistance.

All statistical results are reported as presented in the source materials, with special attention paid to whether outcomes were from interim or final analyses and whether p-values crossed prespecified statistical boundaries.

## 3 Results

### 3.1 Study selection

The results of the systematic literature search and study selection process are summarized in the PRISMA 2020 flow diagram (**Figure 1**). The initial searches across conference proceedings, electronic databases, and hand-searching yielded 93 records. After removal of 19 duplicates using automated and manual methods, 74 unique records underwent title and abstract screening. Of these, 42 records were deemed potentially relevant and proceeded to full-text review. The full-text reports of the 42 records were retrieved and assessed in detail against the eligibility criteria. Forty studies met all inclusion criteria and were included in the qualitative synthesis. The distribution by primary disease site was: bladder cancer (n=13), kidney cancer (n=9), prostate cancer (n=16), testicular cancer (n=1), and penile cancer (n=1).

### 3.2 Study characteristics

**Table 1** summarizes a broad set of genitourinary cancer studies (*n* = 40) presented at ASCO and ESMO 2025 across bladder, kidney, prostate, testicular, and penile cancers. The studies cover multiple clinical settings, including non-muscle invasive and muscle-invasive bladder cancer, metastatic urothelial carcinoma, localized and metastatic prostate cancer, renal cell carcinoma, and rarer malignancies. Study designs were heterogeneous and included randomized phase III trials, randomized phase II trials, single-arm studies, biomarker-guided analyses, and meta-analyses. While many trials were randomized with active comparators, several were non-randomized or single-arm studies. The reported endpoints varied widely across trials and included overall survival (OS), progression-free survival (PFS), disease-free survival (DFS), event-free survival (EFS), pathologic complete response (pCR), metastasis-free survival (MFS), and quality-of-life outcomes. The investigated treatments illustrate the diversity of contemporary therapeutic strategies, including immune checkpoint inhibitors, ADCs, targeted therapies, radioligand therapy, and novel immunotherapeutic approaches.

**Table 1:** Systematic overview of 40 genitourinary cancer studies presented at ASCO and ESMO 2025.

Tumor type	Disease setting	Study name [ref]	Phase [meeting, P]	Design and key eligibility	Intervention	Comparator	Primary endpoint(s) result	Key safety findings (grade ≥3 AEs)
Bladder (13)	NMIBC, High-risk	POTOMAC (1)	III [ESMO, Lancet]	Randomized, double-blind	Durvalumab + BCG	BCG + Placebo	DFS: HR 0.68 (95% CI 0.50-0.93); p=0.0154	Immune-mediated AEs: 8%
	NMIBC, High-risk	ALBAN (2)	III [ESMO, Annals of Oncology]	Randomized	Atezolizumab + BCG	BCG	DFS: HR 0.98 (95% CI 0.71-1.36); p=0.91 (NS)	Immune-mediated AEs: 5.5%
	MIBC, Staging	BladderPath (3)	III [ESMO]	Randomized	Upfront MRI pathway	Standard TURBT pathway	BCSS: HR 0.36 (95% CI 0.12-0.98); p=0.046	NR
	MIBC, Perioperative	NIAGARA (4)	III [ASCO]	Randomized, open-label	Durvalumab + NAC → Adjuvant Durvalumab	NAC alone	EFS: HR 0.68 (95% CI 0.56-0.82); p<0.001	Immune-mediated AEs: 8%
	MIBC, Cisplatin-Ineligible, Perioperative	KEYNOTE-905/EV-303 (5)	III [ASCO, NEJM]	Randomized, open-label	EV + Pembro (neoadj → adj)	Surgery alone	EFS: HR 0.40 (95% CI 0.28-0.57); pCR: 57.1% vs. 8.6%	G3/4 AEs: 71.3% vs. 45.9%
	MIBC, Neoadj	GDFather-NEO (6)	II [ESMO]	Randomized, DB	Nivo + Visugromab (anti-GDF15)	Nivo + Placebo	pCR: 33.3% vs. 7.1%. MPR: 66.7% vs 21.4%.	G3/4 AEs: 12.5% vs. 6.7%
	MIBC, Neoadj (Cisplatin-Ineligible)	SunRISe-4 (SR-4) (7)	II [ESMO]	Randomized, open-label, parallel cohort	TAR-200 (intravesical gemcitabine) + Cetrelimab	Cetrelimab alone	pCR rate: 38% vs. 28%. pOR (≤ypT1): 53% vs. 44%. 1-y RFS: 77% vs. 64%.	No new safety signals. utDNA clearance strongly correlated with pCR (p<10^-5^).
	UTUC, Nephron-sparing	DISTINCT-I (8)	II [ESMO]	Single-arm, HER2-targeted	DV + Tislelizumab → kidney-sparing surgery	None	1-year KI-EFS: 70%, cCR after 4 months 75%	No grade ≥3 systemic toxicities
	mUC, 1L	KEYNOTE-A39/EV-302 (9)	III [ESMO, Lancet Oncology]	Randomized, open-label	Enfortumab vedotin + Pembrolizumab	Gem/Cis or Gem/Carbo	OS: HR 0.47; p<0.001 PFS: HR 0.45; p<0.001	Grade ≥3 AEs in 55.9% of pts: rash (7.7%), hyperglycemia (5%).
	mUC, 1L Cisplatin-Ineligible	CheckMate 901 (10)	III [ASCO]	Randomized	Nivolumab + Ipilimumab	Gem/Carbo	OS: Superior with N+I	Treatment-related AEs consistent with dual IO
	mUC, 1L, HER2+	RC48-C016 (11, 12)	III [ESMO, NEJM]	Randomized, open-label	Disitamab Vedotin + Toripalimab	Gem/Cis or Gem/Carbo	PFS: HR 0.36; OS: HR 0.54	Any G3/4 55,1% vs. 86.9%
	mUC, 1L Maintenance	JAVELIN Bladder Medley (13)	II [ASCO]	Randomized, open-label	Avelumab + Sacituzumab Govitecan	Avelumab	PFS: Improved with combination	SG-related toxicities (diarrhea, neutropenia)
	mUC, 1L	DISCUS (14)	II [ESMO, Annals of Oncology]	Randomized	3 cycles chemo → Avelumab	6 cycles chemo → Avelumab	QoL: +8.5 pts (p=0.016). OS: 18.9 mo both arms (HR 1.15). PFS: 8.0 vs. 9.0 mo (HR 1.05).	TRAEs: 6% vs. 11%
Kidney (9)	RCC, Adjuvant	RAMPART (15)	III [ESMO]	3-arm, randomized	Arm C: Durva + Treme → Durva; Arm B: Durva → Durva	Arm A: Active Monitoring	DFS (Arm C): HR 0.65	G3/4 AEs: Arm C 40%, Arm A 8%
	RCC, Neoadj	NESCIO (16)	II [ESMO]	Randomized, non-comparative, 3-arm	A: Nivo; B: Ipi+Nivo; C: Nivo+Relatlimab	Arms not compared	Pathologic Response: A: 7.1%; B: 14.3%; C: 14.3%	Immune-related AEs: A: 6.7%; B: 42.8%; C: 14.2%
	mccRCC, 1L	CheckMate 214 Final (17)	III [ASCO]	Randomized	Nivolumab + Ipilimumab	Sunitinib	5-year OS: 48% vs. 37% (HR 0.72)	G3/4 TRAEs: 48% vs. 64%
	mccRCC, 1L	OPTIC RCC (18)	II [ESMO]	Biomarker-stratified	RNA-seq guided therapy	Non-randomized	ORR (Angio/Stromal): 71%	-
	mccRCC	PDIGREE Step 1 (19)	III [ASCO]	Adaptive, randomized	Epi+Nivo → Nivo vs. Cabo+/-Nivo	Adaptive	Step 1 efficacy and safety outcomes	G3/4 AEs: Diarrheas (5%, AST/ALT elevation (6%))
	RCC, Post-VEGF	CALYPSO (20)	II [ESMO]	Randomized	Durvalumab ± Savolitinib ± Tremelimumab	Durvalumab monotherapy	cRR: D = 11%, DT = 33%; OS: D = 25.8 mo; DT = 24.2 mo, DS = 16.3 mo	G3/4 AEs: D 10%, DT 23%, DS 23%
	mccRCC, Post-IO	LenCabo (21)	II [ESMO, Annals of Oncology]	Randomized	Lenvatinib + Everolimus	Cabozantinib	PFS: HR 0.51 (95% CI 0.29-0.89)	Hypertension, proteinuria
	mccRCC, 2L+	FRUSICA-2 (22)	II/III [ESMO]	Randomized, open-label	Fruquintinib + Sintilimab	Axtinib or Everolimus	PFS: 22.21 vs. 6.90 mo (HR 0.373; p<0.0001) ORR (60.5% vs. 24.3%)	Hypertension (12%), proteinuria (4%)
	tRCC	AREN1721 (23)	II [ASCO]	Randomized	Axitinib + Nivolumab	Nivolumab	PFS: 10 vs. 4 mo, p = 0.00058	Hypertension (42%), fatigue (28%)
Prostate (16)	Localized, High-risk	ENZARAD (24)	III [ESMO]	Randomized	Enzalutamide + ADT + RT	Placebo + ADT + RT	MFS: HR 0.88 (95% CI 0.67-1.15); p=0.34 (NS)	Fatigue G3+ (16% vs. 6%), Nervous system 25% vs. 15%Hypertention (34% vs. 25%)
	Localized PCa	CAN-2409 + EBRT (25)	III [ASCO]	Randomized, placebo-controlled	CAN-2409 + prodrug + SOC EBRT +/- ADT	Placebo + SOC EBRT +/- ADT	Improved prostate cancer-specific DFS: NR vs. 86.1 mo, HR 0.7. p=0.015	Generally well tolerated
	High-risk non-metastatic PCa	STAMPEDE MMAI Model (26)	III [ASCO, Lancet Digital Health]	AI biomarker analysis	MMAI-guided ARPI selection	Standard selection	MMAI identifies differential ARPI benefit	N/A
	BCR, High-risk	EMBARK (27)	III [ESMO]	Randomized, double-blind	Enzalutamide + Leuprolide	Leuprolide + Placebo	OS: HR 0.597 (95% CI 0.444-0.804)	G3/4 AEs: 52% vs. 49.4
	BCR, High-risk	PRESTO (28)	III [ESMO]	Randomized, open-label	ADT + Apalutamide (± Abiraterone)	ADT alone	MFS (RMST): +2.92 mo for ADT+APA vs. ADT	Consistent with known profiles
	Oligometastatic PCa	Metacure B2/B2 Expansion (29)	II [ASCO]	Single-arm cohorts	Intensified hormonal blockade + SBRT	None	12-month PSA ≤0.2 ng/mL	In line with expected toxicity
	mHSPC	ARCHES 5-Year Follow-up (30)	III [ASCO]	Long-term follow-up	Enzalutamide + ADT	Placebo + ADT	5-year OS benefit maintained	TEAEs diminished over time
	mHSPC, HRR-altered	AMPLITUDE (31)	III [ASCO]	Randomized	Niraparib + AAP	Placebo + AAP	rPFS: HR 0.72 (95% CI 0.58-0.89)	Anemia (30%), thrombocytopenia (15%)
	mHSPC, PTEN-deficient	CAPItello-281 (32)	III [ESMO, Annals of Oncology]	Randomized	Capivasertib + Abiraterone	Placebo + Abiraterone	rPFS: HR 0.81 (95% CI 0.67-0.98)	Rash (28%), diarrhea (22%)
	mHSPC (Meta-analysis)	STOPCAP (33)	Meta-analysis [ESMO]	IPD from RCTs	Prostate RT + Systemic Therapy	Systemic Therapy alone	OS in low-volume: HR 0.79 (95% CI 0.67-0.93); p=0.005	NR
	mHSPC	ARASAFE (34)	III [ESMO]	Randomized, non-inferiority	2-weekly Docetaxel (50 mg/m²) + Darolutamide + ADT	3-weekly Docetaxel (75 mg/m²) + Darolutamide + ADT	Febrile neutropenia significantly lower. Efficacy (Secondary): PSA response ≥90% at 6 months comparable.	Febrile neutropenia reduced; other key toxicities (e.g., fatigue, neuropathy) manageable.
	mHSPC	ARANOTE HRQoL Analysis (35)	III [ASCO]	HRQoL analysis	Darolutamide + ADT	Placebo + ADT	Delayed pain progression, preserved QoL	Favorable HRQoL profile
	mHSPC, PSMA+	PSMAddition (36)	III [ESMO]	Randomized, open-label	[^177^Lu]Lu-PSMA-617 (7.4 GBq q6w × 6 cycles) + ADT + ARPI	ADT + ARPI alone	rPFS: HR 0.72 (95% CI 0.58-0.90); p=0.002 (primary). ORR: 85.3% vs. 80.8%	G3/4 AEs: 31.9% vs. 28.7%; Dry mouth (grade 1-2): 45.8% vs. 3.8%; Grade ≥3 cytopenias: 14.4% vs. 5.0%
	mCRPC, HRR-deficient	TALAPRO-2 Exploratory (37)	III (Exploratory) [ASCO]	Post-hoc biomarker analysis	Talazoparib + Enza	Placebo + Enza	rPFS, OS by HRR gene subgroup	N/A
	mCRPC, ARPI-naïve	PETRANHA (38)	Ib/II [ASCO]	Single-arm	Saruparib + ARPI	None	PSA90: 68% in ARPI-naïve cohort	Anemia (19.5%), fatigue (1.3%)
	Heavily pretreated mCRPC	Pasritamig (39)	I [ASCO, JCO]	First-in-human	Pasritamig (KLK2xCD3 TCE)	None	PSA50: 42.4% at RP2D	Favorable with low TRAEs, minimal CRS
Testicular (1)	Seminoma IIA/B	SAKK 01/10 (40)	II [ESMO]	Single-arm	Single-dose Carboplatin + Involved-node RT	Historical	10-year PFS: 92.8%	Minimal long-term toxicity; 1 thromboembolic event
Penile (1)	High-risk, post-resection	Adjuvant Chemo Comparison (41)	III [ASCO]	Randomized, non-inferiority	Platinum + Paclitaxel	Platinum + 5-FU	OS (37.2 vs. 21.6 mo, p=0.530)	Higher G3/4 hematologic and GI toxicities

AE, adverse event; ADT, androgen deprivation therapy; ARPI, androgen receptor pathway inhibitor; BCG, Bacillus Calmette-Guérin; BCR, biochemical recurrence; BID, twice daily; CI, confidence interval; CR, complete response; CRS, cytokine release syndrome; DFS, disease-free survival; DoR, duration of response; EBRT, external beam radiotherapy; EFS, event-free survival; EV, enfortumab vedotin; G3/4, grade 3 or 4; HR, hazard ratio; HRQoL, health-related quality of life; HRR, homologous recombination repair; IO, immunotherapy; Ipi, ipilimumab; IV, intravenous; MFS, metastasis-free survival; MIBC, muscle-invasive bladder cancer; mCRPC, metastatic castration-resistant prostate cancer; mHSPC, metastatic hormone-sensitive prostate cancer; mUC, metastatic urothelial carcinoma; NAC, neoadjuvant chemotherapy; Nivo, nivolumab; NMIBC, non-muscle-invasive bladder cancer; NS, not significant; ORR, objective response rate; OS, overall survival; pCR, pathologic complete response; Pembro, pembrolizumab; PFS, progression-free survival; PR, partial response; PSA, prostate-specific antigen; PSMA, prostate-specific membrane antigen; Q2W, every 2 weeks; Q3W, every 3 weeks; Q4W, every 4 weeks; QoL, quality of life; RCC, renal cell carcinoma; RCT, randomized controlled trial; RNA-seq, RNA sequencing; RP2D, recommended phase 2 dose; rPFS, radiographic progression-free survival; RT, radiotherapy; SOC, standard of care; TEAE, treatment-emergent adverse event; TRAE, treatment-related adverse event; TURBT, transurethral resection of bladder tumor; UTUC, upper tract urothelial carcinoma.

### 3.3 Risk of bias assessment results

A methodological appraisal of the included studies was performed using the Cochrane risk-of-bias framework for randomized trials, while non-randomized and single-arm studies were assessed descriptively due to the limitations of applying RoB 2 in such designs. The overall body of evidence included a mixture of phase III randomized trials, phase II randomized studies, and several early-phase or single-arm investigations. Randomized phase III trials generally exhibited more robust methodological features, whereas open-label designs and non-randomized studies inherently carried a higher potential for bias. Taken together, the evidence base reflects a heterogeneous methodological landscape, ranging from large confirmatory trials to exploratory early-phase studies evaluating emerging therapeutic strategies.

### 3.4 Thematic synthesis of findings by tumor site and stage

#### 3.4.1 Bladder cancer

##### 3.4.1.1 Non-metastatic disease (curative-intent)

###### 3.4.1.1.1 Non-muscle-invasive bladder cancer, high-risk

The POTOMAC trial was a phase III, randomized, double-blind study that evaluated the addition of durvalumab (an anti-PD-L1 antibody) to standard Bacillus Calmette-Guérin (BCG) therapy in BCG-naive patients with high-risk non-muscle-invasive bladder cancer (NMIBC) NMIBC. The study enrolled approximately 800 patients who were randomized to receive either durvalumab plus BCG or placebo plus BCG. The primary endpoint was disease-free survival (DFS) (1). The results demonstrated a statistically significant improvement in DFS with the durvalumab combination, with a hazard ratio of 0.68 (95% CI 0.50-0.93; p=0.0154). Subgroup analyses showed consistent benefit across key patient characteristics, including age, tumor stage, and prior resection status. The safety profile was manageable, with immune-mediated AEs occurring in 27% of patients in the durvalumab arm, primarily consisting of grade 1–2 events. Grade 3 or higher immune-related AEs were reported in 8% of patients.

**Figure 2:**
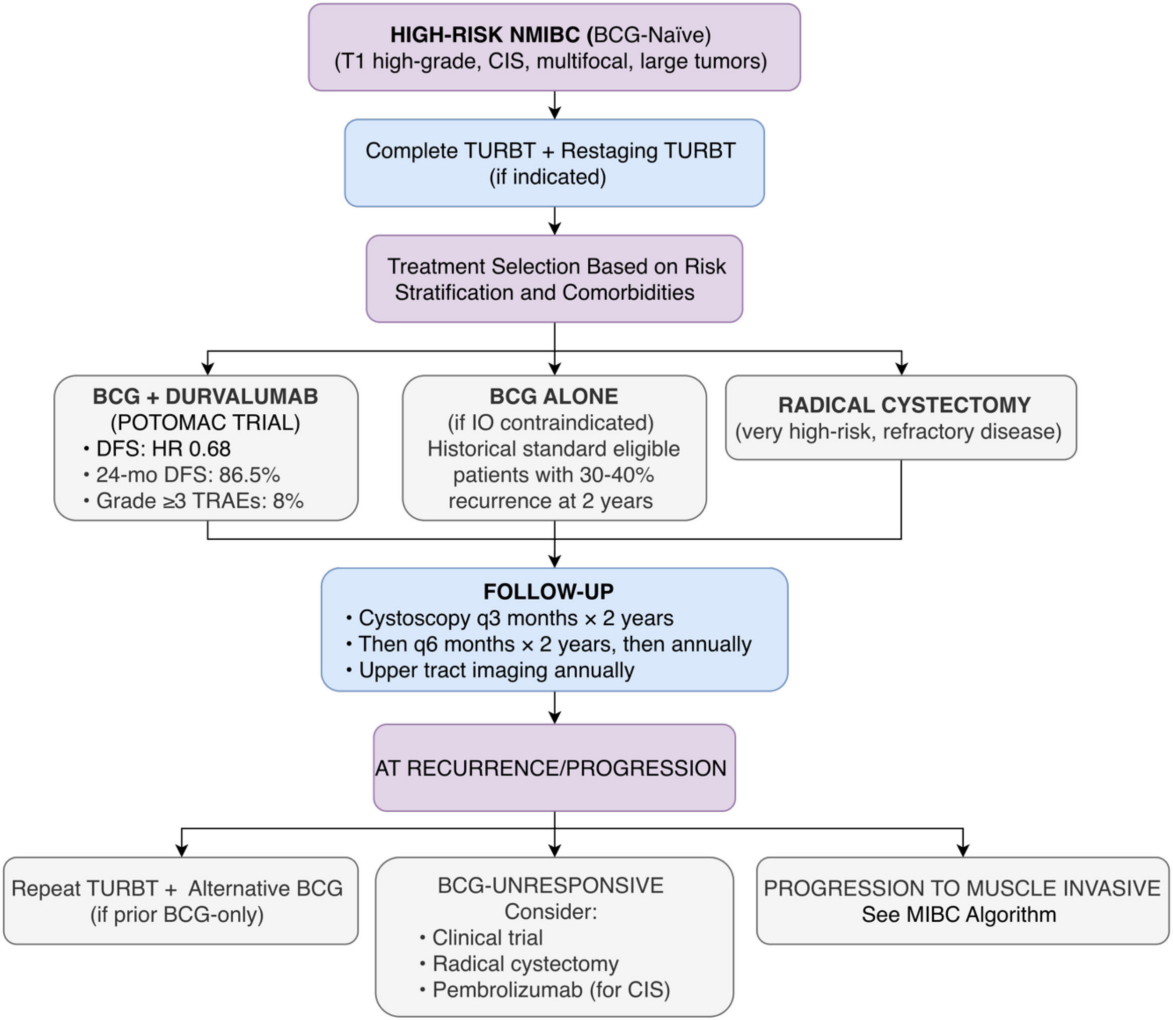
Proposed treatment algorithm for high-risk non-muscle-invasive bladder cancer (NMIBC) based on 2025 ASCO/ESMO data. This diagram integrates findings from the POTOMAC (positive) and ALBAN (negative) Phase III trials of PD-(L)1 inhibitors combined with BCG. The algorithm proposes durvalumab plus BCG as a potential new option for eligible patients, while acknowledging BCG alone remains appropriate for those with immunotherapy contraindications. Caution: This is a proposed framework; POTOMAC requires regulatory approval, and longer follow-up is needed.

In contrast, the ALBAN trial, another phase III study, evaluated atezolizumab (an anti-PD-L1 antibody) in combination with BCG versus BCG alone in a similar patient population (2). This trial enrolled patients with high-risk NMIBC who were BCG-naive. The primary endpoint was also DFS. The results showed no statistically significant difference in DFS between the two arms, with a hazard ratio of 0.98 (95% CI 0.71-1.36; p=0.91). The safety profile was similar to that observed with other immune checkpoint inhibitors, with grade 3 or higher AEs of 5.5%. The divergent outcomes between POTOMAC (positive) and ALBAN (negative) highlight potential differences between PD-1/PD-L1 inhibitors in this setting and underscore the need for further biomarker exploration to identify patients most likely to benefit from immunotherapy in NMIBC. A proposed algorithm for managing high-risk NMIBC based on the divergent results of POTOMAC and ALBAN is shown in **Figure 2**.

###### 3.4.1.1.2 Muscle-invasive bladder cancer, diagnostic staging

The BladderPath trial established upfront MRI as a superior alternative to traditional transurethral resection (TURBT) for staging bladder cancer. In this randomized study, the MRI-based pathway significantly improved bladder cancer-specific survival, reducing the risk of death by 64% compared to initial TURBT (HR 0.36; 95% CI 0.12–0.98; p=0.046). By enabling earlier, non-invasive, and more accurate identification of muscle-invasive disease, upfront MRI staging represents a new evidence-based standard that can expedite appropriate treatment and improve outcomes (3).

###### 3.4.1.1.3 Muscle-invasive bladder cancer, perioperative setting

The NIAGARA trial was a global, phase III, randomized study that investigated the addition of perioperative durvalumab to neoadjuvant chemotherapy in patients with MIBC who were eligible for cisplatin-based chemotherapy (4). The trial enrolled approximately 1,000 patients, who were randomized to receive either durvalumab plus chemotherapy, followed by adjuvant durvalumab, or chemotherapy alone, followed by observation. The primary endpoint was event-free survival (EFS). The results demonstrated a significant improvement in EFS with the durvalumab-containing regimen, with a hazard ratio of 0.68 (95% CI 0.56-0.82; p<0.001). Subgroup analyses based on circulating tumor DNA (ctDNA) status showed that benefit was observed regardless of baseline ctDNA status, but patients with ctDNA clearance during treatment had superior outcomes. The safety profile showed an increase in immune-mediated AEs with durvalumab (20.9% any grade, 8% grade 3-4), but no new safety signals were identified.

**Figure 3:**
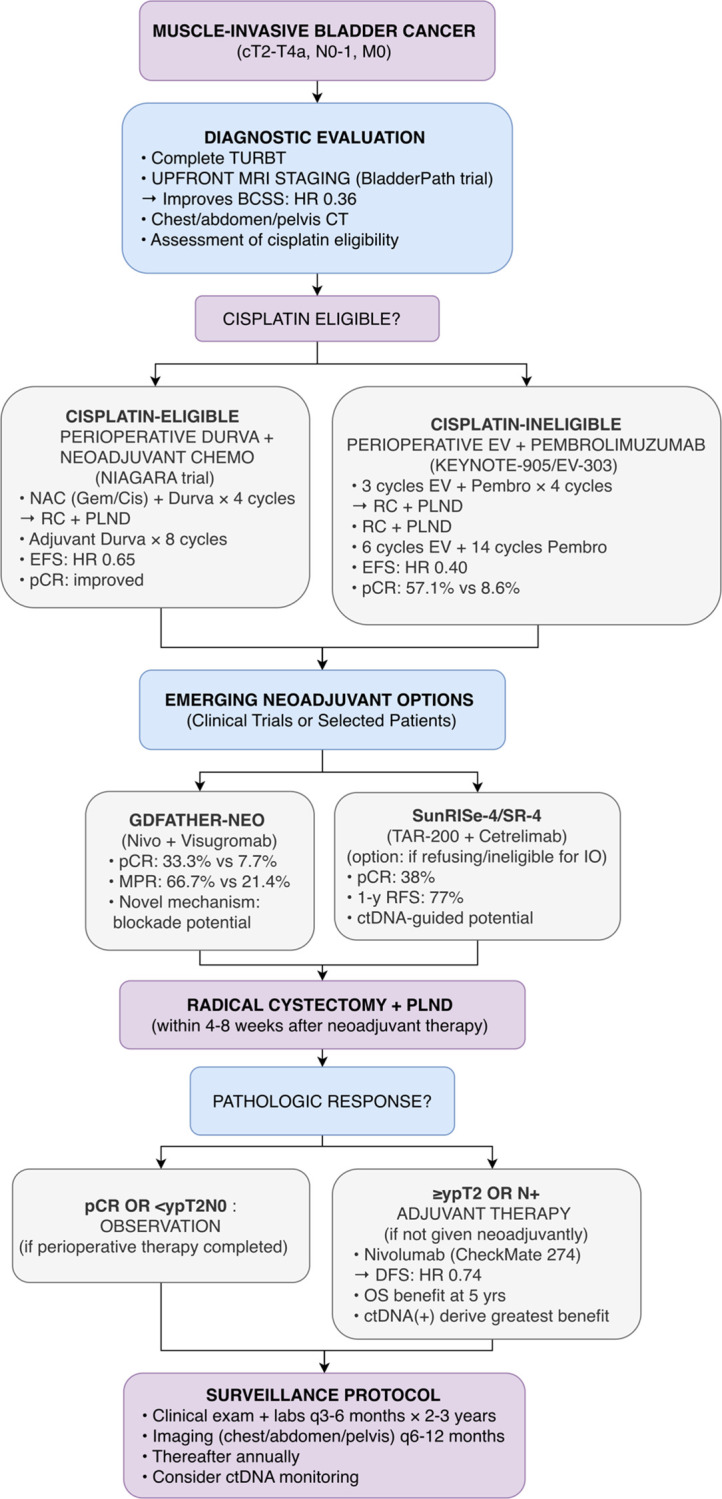
Proposed treatment algorithm for non-metastatic muscle-invasive bladder cancer (MIBC) based on 2025 ASCO/ESMO data. This algorithm incorporates upfront MRI staging (BladderPath Phase III), perioperative durvalumab for cisplatin-eligible patients (NIAGARA Phase III), and perioperative enfortumab vedotin plus pembrolizumab for cisplatin-ineligible patients (KEYNOTE-905/EV-303 Phase III). Emerging neoadjuvant options from Phase II trials (GDFather-NEO, SunRISe-4) are shown as investigational. Adjuvant nivolumab is included based on CheckMate 274 5-year update. Caution: Phase II results require confirmation; regulatory approvals pending for several agents.

The KEYNOTE-905/EV-303 trial addressed the unmet need in cisplatin-ineligible patients with MIBC (5). This phase III, randomized study evaluated perioperative enfortumab vedotin (an ADC targeting Nectin-4) plus pembrolizumab versus surgery alone in cisplatin-ineligible patients. The trial enrolled approximately 700 patients. The primary endpoints were EFS and pCR rate. The results showed a dramatic improvement in both endpoints: EFS hazard ratio was 0.40 (95% CI 0.28-0.57; p<0.0001), and pCR rate was 57.1% in the experimental arm versus 8.6% in the control arm. The safety profile was characterized by expected toxicities of enfortumab vedotin, including rash (25.1% any grade) and pruritus (47.7% any grade), but these were manageable with appropriate supportive care and dose modifications. **Figure 3** integrates perioperative options for MIBC, including the paradigm-shifting results of NIAGARA and KEYNOTE-905/EV-303.

The phase II GDFather-NEO trial is a double-blind, randomized study based on the recent identification of growth and differentiation factor 15 (GDF-15) as a key mediator of resistance to PD-(L)1 blockade. The trial evaluated the addition of visugromab, a GDF-15-neutralizing antibody, to nivolumab compared to nivolumab plus placebo, administered for three cycles prior to surgery. The results were striking: the combination tripled the pCR rate (33.3% vs. 7.1%) and significantly increased the major pathologic response (MPR) rate (66.7% vs. 21.4%). Importantly, the regimen demonstrated excellent tolerability, with no new safety signals identified. This study provides the first clinical proof-of-concept that targeting the GDF-15 pathway can dramatically enhance the efficacy of anti-PD-1 therapy, offering a promising new avenue for treatment intensification in MIBC and supporting further development in larger, response-adaptive bladder-preservation studies (6).

The phase II SunRISe-4 trial (SR-4) evaluated a novel bladder-sparing strategy in patients with MIBC who were ineligible for or refused platinum-based neoadjuvant chemotherapy. This randomized study compared neoadjuvant intravesical gemcitabine (TAR-200) combined with the PD-1 inhibitor cetrelimab versus cetrelimab alone. The combination arm demonstrated a higher pCR rate of 38% compared to 28% with cetrelimab monotherapy, along with improved pathologic overall response (≤ypT1) and 1-year recurrence-free survival rates. Exploratory biomarker analysis revealed that clearance of urinary tumor DNA (utDNA) during treatment was significantly associated with achieving pCR, highlighting its potential as a dynamic, predictive biomarker. These results support the further investigation of combined local and systemic immunotherapy as a potent neoadjuvant regimen for cisplatin-ineligible MIBC (7).

###### 3.4.1.1.4 Upper tract urothelial carcinoma

The phase II DISTINCT-I trial evaluated a kidney-preserving paradigm for patients with high-risk upper tract urothelial carcinoma (UTUC) who had an imperative indication for renal preservation (solitary kidney or baseline eGFR <60 mL/min/1.73 m²). This novel strategy combined perioperative anti-HER2 ADC (disitamab vedotin) with the PD-1 inhibitor tislelizumab, followed by kidney-sparing surgery (endoscopic ablation or ureteral segmental resection). In 19 evaluable patients, the regimen demonstrated a 1-year kidney-intact EFS (KI-EFS) rate of 70% and induced a clinical complete response (cCR) rate of 75% after 4 months. Notably, no grade ≥3 systemic toxicities were observed. While HER2 overexpression (IHC 3+/2+) was present in 45% of patients and may correlate with response, these results challenge the historical standard of mandatory radical nephroureterectomy, offering a promising function-preserving approach for selected high-risk UTUC patients (8).

##### 3.4.1.2 Advanced/metastatic disease

###### 3.4.1.2.1 First-line therapy

The phase III KEYNOTE-A39/EV-302 trial established enfortumab vedotin plus pembrolizumab (EV+P) as the new frontline standard for mUC. Compared to platinum-based chemotherapy in 1,200 treatment-naive patients, EV+P demonstrated unprecedented efficacy. The initial analysis showed greater than 50% reductions in the risk of death and progression (OS HR = 0.47; PFS HR = 0.45), with a doubled complete response rate of 29.1%. A 2.5-year median follow-up update confirmed the durability of this benefit, revealing a median OS of 33.8 months versus 15.9 months with chemotherapy (HR 0.51) and a median PFS of 12.5 months versus 6.3 months (HR 0.48). This long-term survival data is particularly significant. Despite a distinct toxicity profile including rash, and hyperglycemia, these AEs have proven manageable in clinical practice and did not compromise the major survival benefit. These robust results definitively solidify EV+P as the standard of care in the first-line setting, irrespective of cisplatin eligibility or PD-L1 status (9).

The CheckMate 901 trial provided an alternative frontline option for cisplatin-ineligible patients (10). This phase III, randomized study compared nivolumab plus ipilimumab versus gemcitabine plus carboplatin in cisplatin-ineligible patients with mUC. The trial enrolled approximately 600 patients. The primary endpoint was OS, which was improved with the immunotherapy combination (hazard ratio 0.79; 95% CI 0.61-1.01; p=0.0245). This offers a chemotherapy-free option for patients who cannot tolerate platinum-based chemotherapy (10).

**Figure 4:**
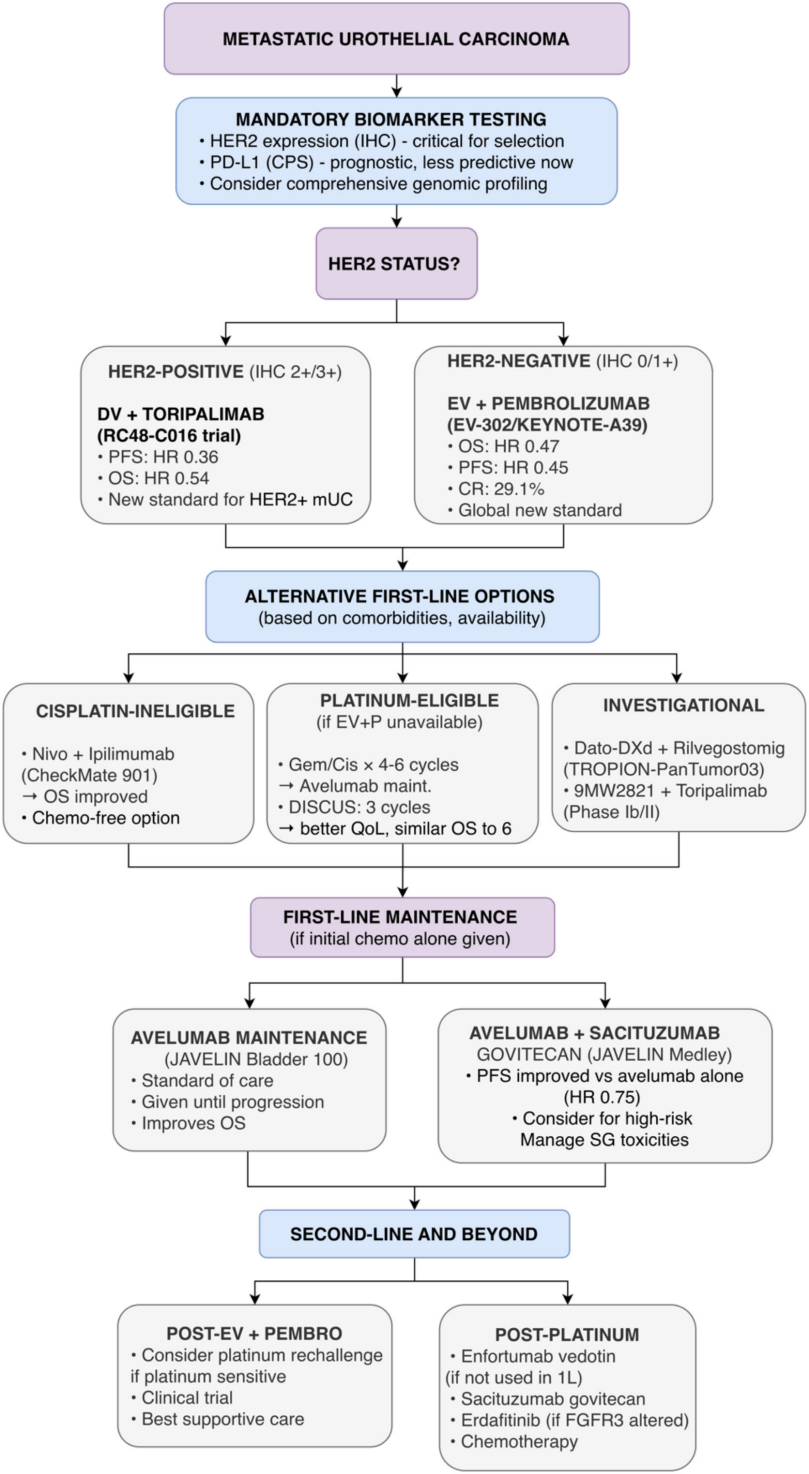
Proposed treatment algorithm for metastatic urothelial carcinoma (mUC) based on 2025 ASCO/ESMO data. This biomarker-driven algorithm mandates HER2 testing at baseline. For HER2-positive patients, disitamab vedotin plus toripalimab is proposed based on Phase III RC48-C016. For HER2-negative patients, enfortumab vedotin plus pembrolizumab is the new standard from EV-302. Alternative options include nivolumab plus ipilimumab (CheckMate 901) and abbreviated chemotherapy followed by avelumab (DISCUS Phase II). Later-line options include FGFR3 inhibition (LY3866288, FORAGER-1) and investigational ADC combinations (TROPION-PanTumor03). Caution: DISCUS and FORAGER-1 are Phase II; RC48-C016 conducted in China; global applicability requires confirmation.

The RC48-C016 trial established a biomarker-defined frontline standard for HER2-positive mUC (11, 12). This phase III, randomized study compared disitamab vedotin plus toripalimab versus platinum-based chemotherapy in patients with HER2-positive (IHC 2+/3+) mUC. The trial enrolled approximately 500 patients. The dual primary endpoints were PFS and OS. The results showed dramatic improvement in both endpoints: PFS hazard ratio was 0.36 (95% CI 0.28-0.46; mPFS 13.1 vs. 6.5 months, p<0.001), and OS hazard ratio was 0.54 (95% CI 0.41-0.73; mOS 31.5 vs. 16.9 months, p<0.001). This establishes a new standard for the HER2-positive subset and underscores the importance of HER2 testing in mUC. The biomarker-driven approach to metastatic urothelial carcinoma, with HER2 status directing therapy selection, is illustrated in **Figure 4**.

###### 3.4.1.2.2 Maintenance therapy post-first-line chemotherapy

The JAVELIN Bladder Medley trial explored combination maintenance therapy after first-line chemotherapy (13). This phase II, randomized study compared avelumab plus sacituzumab govitecan (an anti-TROP2 ADC) versus avelumab alone in patients who had not progressed after 4–6 cycles of platinum-based chemotherapy. The trial enrolled approximately 200 patients. The primary endpoint was PFS, which was improved with the combination (HR 0.49; 95% CI 0.31-0.76). Notably, the combination doubled the median PFS compared to avelumab monotherapy. The safety profile included expected toxicities of sacituzumab govitecan, particularly grade ≥3 diarrhea (12%) and neutropenia (39.7%), but these were manageable with dose adjustments and supportive care.

Concurrently, the phase II DISCUS trial randomized 267 patients with untreated mUC to receive either three (3C) or six (6C) cycles of gemcitabine plus cisplatin/carboplatin, each followed by avelumab maintenance, to evaluate whether a shorter chemotherapy duration could improve quality of life (QoL) without compromising efficacy (14). Co-primary endpoints were patient-reported global health status/QoL at cycle 6 and OS. The mean QoL change significantly favored the 3C arm (+8.5 points, 95% CI 0.7–16.3, p=0.016). Interim OS was identical in both arms (18.9 months; HR 1.15, 95% CI 0.72–1.86), and mPFS was 8.0 versus 9.0 months (HR 1.05, 95% CI 0.73–1.53). Grade 3–4 treatment-related adverse events (TRAEs) were lower with three cycles (6% vs. 11%). The study concluded that 3C of chemotherapy followed by avelumab maintenance resulted in significantly better QoL with comparable survival outcomes, demonstrating the feasibility of patient-centered, shorter-duration regimens.

#### 3.4.2 Kidney cancer

##### 3.4.2.1 Neo/adjuvant therapy

The evolving role of immunotherapy in non-metastatic renal cell carcinoma (RCC) is being defined by trials in both the adjuvant and neoadjuvant settings. The phase III RAMPART trial established a significant advance in adjuvant therapy, demonstrating that one year of durvalumab in combination with tremelimumab significantly improves DFS compared to active monitoring in high-risk patients post-nephrectomy (15). The hazard ratio was 0.65 for the combination, with a more modest benefit for durvalumab monotherapy (HR 0.77). The benefit was most pronounced in the highest-risk subgroup (HR 0.52). This efficacy came with a defined toxicity trade-off, as grade 3–4 TRAEs occurred in 28% and 42% of patients in the monotherapy and combination arms, respectively.

Concurrently, the phase II NESCIO trial explored the feasibility and activity of a neoadjuvant approach in high-risk, resectable clear cell RCC (16). This randomized study tested short-course (6-week) therapy with nivolumab monotherapy, ipilimumab plus nivolumab, or nivolumab plus relatlimab. Pathologic response rates were modest (7.1% to 14.3%), but the study crucially illuminated the risk-benefit calculus: the ipilimumab-nivolumab arm showed slightly higher activity but was associated with a substantially higher rate of grade ≥3 immune-related AEs (42.8%).

**Figure 5:**
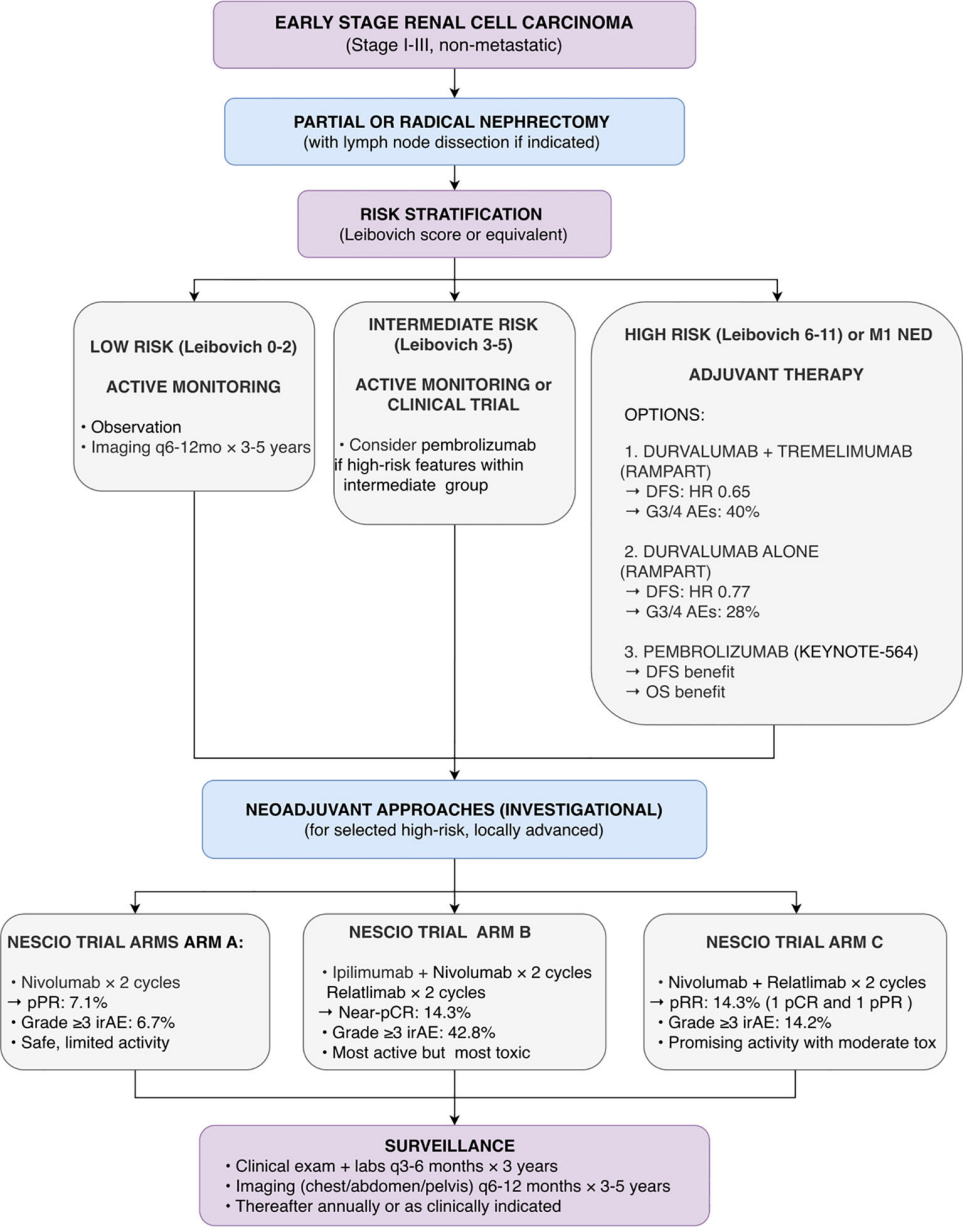
Proposed treatment algorithm for localized renal cell carcinoma (RCC) based on 2025 ASCO/ESMO data. Following nephrectomy, patients are stratified by Leibovich risk. For high-risk patients, adjuvant options from the Phase III RAMPART trial are shown: durvalumab plus tremelimumab (DFS HR 0.65) or durvalumab monotherapy (DFS HR 0.77). Pembrolizumab remains an alternative based on KEYNOTE-564. Neoadjuvant immunotherapy combinations from the Phase II NESCIO trial are shown as investigational. Caution: RAMPART OS data immature; NESCIO Phase II results require validation; optimal adjuvant regimen unclear.

Collectively, the RAMPART and NESCIO trials affirm the biological activity of immune checkpoint inhibitors in non-metastatic RCC while powerfully underscoring the critical need for predictive biomarkers to guide patient selection, optimize therapeutic intensity, and balance meaningful clinical benefit with treatment-related morbidity. Adjuvant options from RAMPART and investigational neoadjuvant approaches from NESCIO are synthesized in **Figure 5**.

##### 3.4.2.2 First-line metastatic clear cell RCC

The final analysis of CheckMate 214 with 7.5 years of follow-up confirmed the durable benefit of nivolumab plus ipilimumab in intermediate/poor-risk metastatic clear cell RCC (17). This phase III, randomized study compared nivolumab plus ipilimumab versus sunitinib. The long-term results showed 5-year OS rates of 48% versus 37% (hazard ratio 0.72; 95% CI 0.62-0.84), confirming the durability of response with the immunotherapy combination. The safety profile showed that while grade 3–4 TRAEs were higher with the combination during treatment (48% vs. 64%), late toxicities were rare and manageable.

The OPTIC RCC trial introduced a novel transcriptomic phenotype-guided approach to first-line therapy (18). This phase II, biomarker-stratified study used RNA sequencing to classify patients into Angiogenic/Stromal or Immune clusters, then assigned treatment accordingly: Angiogenic/Stromal patients received nivolumab plus cabozantinib, while Immune patients received ipilimumab plus nivolumab. The primary endpoint was ORR, which was 71% in the Angiogenic/Stromal cluster. This proof-of-concept study demonstrates the potential of transcriptomic profiling to guide therapy selection beyond clinical risk stratification alone.

The PDIGREE trial is a phase III, adaptive platform study designed to optimize treatment sequencing for intermediate- or poor-risk metastatic clear cell RCC (mccRCC). In its initial step (Step 1), all enrolled patients received first-line ipilimumab plus nivolumab. The study’s adaptive design entailed subsequent treatment based on response at 12 weeks: patients with progressive disease were assigned to cabozantinib monotherapy, while those with non-progressive disease were randomized to receive nivolumab with or without cabozantinib. The presented results from this induction phase provide critical data on the regimen’s tolerability in a large, representative cohort. Among 1,111 treated patients, 33% discontinued therapy during Step 1, with TRAEs being the leading cause (accounting for 44% of all discontinuations). These findings characterize the discontinuation rates associated with first-line ipilimumab-nivolumab in this setting and establish the baseline for the adaptive randomization, which evaluates the role of cabozantinib, in the trial’s subsequent steps (19).

##### 3.4.2.3 Post-immunotherapy sequencing

The optimal sequencing of therapy for advanced RCC following progression on first-line regimens is a critical clinical challenge, addressed by several pivotal 2025 studies in distinct patient populations.

For patients who have progressed on prior VEGF-targeted therapy, the phase II CALYPSO trial evaluated novel combinations of durvalumab, savolitinib (a MET inhibitor), and tremelimumab. While the primary endpoint was not met, the durvalumab plus tremelimumab combination showed a higher confirmed response rate (33%) compared to durvalumab monotherapy (11%). An exploratory biomarker analysis showed no survival benefit in PDL1-positive subgroups (20).

For patients progressing after first-line immunotherapy, the phase II LenCabo trial provided essential head-to-head evidence, comparing lenvatinib plus everolimus versus cabozantinib. The study demonstrated a significantly improved PFS with the lenvatinib-everolimus combination (HR 0.51; 95% CI 0.29–0.89; p=0.02), establishing it as a new potential therapeutic sequence in this setting (21).

Concurrently, the phase II/III FRUSICA-2 trial redefined second-line treatment for patients progressing after a VEGFR-TKI. The study compared fruquintinib (VEGFR-TKI) plus sintilimab (an anti-PD-1 antibody) to investigator’s choice of axitinib or everolimus monotherapy. The combination therapy demonstrated superior efficacy, more than tripling median PFS (22.21 vs. 6.90 months; HR 0.373, p<0.0001) and doubling the ORR (60.5% vs. 24.3%), with a manageable safety profile. This establishes fruquintinib plus sintilimab as a highly effective new standard for second-line RCC (22).

**Figure 6:**
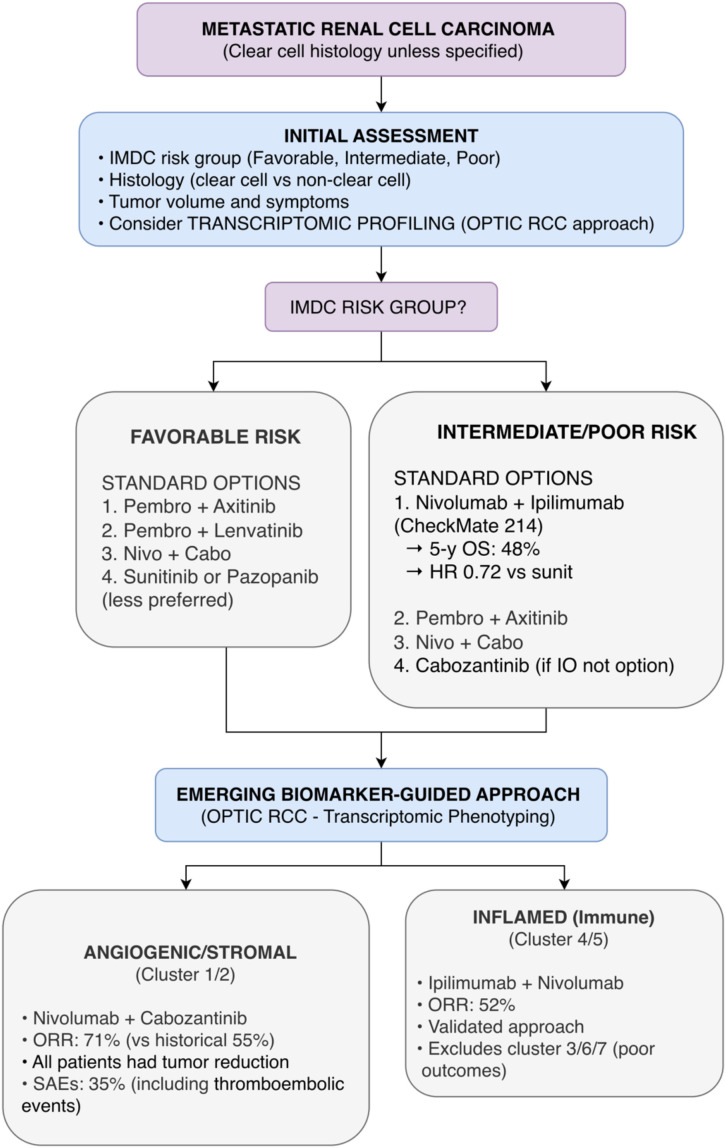
Proposed treatment algorithm for metastatic renal cell carcinoma (mRCC) based on 2025 ASCO/ESMO data. This algorithm integrates IMDC risk stratification with emerging transcriptomic-guided therapy (OPTIC RCC Phase II). Nivolumab plus ipilimumab remains standard for intermediate/poor-risk patients based on CheckMate 214 7.5-year follow-up. For rare subtypes, axitinib plus nivolumab is proposed for translocation RCC (AREN1721 Phase II). Post-progression options include lenvatinib plus everolimus versus cabozantinib (LenCabo Phase II) and fruquintinib plus sintilimab (FRUSICA-2 Phase II/III). Caution: OPTIC RCC, LenCabo, and AREN1721 are Phase II requiring confirmation; FRUSICA-2 combination not globally available.

These trials collectively provide a modern, evidence-based framework for sequencing therapy in advanced RCC. LenCabo defines the preferred regimen (lenvatinib plus everolimus) after immunotherapy progression. FRUSICA-2 establishes a new, highly active standard (fruquintinib plus sintilimab) in the second-line post-VEGF setting. Meanwhile, CALYPSO contributes exploratory data on combination immunotherapy and identifies a potential biomarker-defined subgroup (MET-driven) for future personalized strategies. This evidence enables a more personalized and effective sequencing approach, tailored to the nature of prior treatment and individual patient biomarkers. **Figure 6** presents a proposed algorithm for metastatic RCC incorporating IMDC risk, transcriptomic profiling, and evidence-based sequencing.

##### 3.4.2.4 Rare subtypes

The management of rare RCC subtypes represents a significant unmet need, with translocation RCC (tRCC) being a particularly aggressive variant driven by TFE3 or TFEB gene fusions. The phase II AREN1721 trial addressed this gap by evaluating axitinib plus nivolumab versus nivolumab monotherapy in patients (both pediatric and adult) with advanced tRCC (23). Despite early closure due to poor accrual, a common challenge in rare cancers, the trial yielded practice-informing results. The combination significantly improved PFS, extending the median PFS from 1.8 months with nivolumab alone to 10.5 months (HR 0.52). Furthermore, 33% of patients in the combination arm achieved a partial response, compared to 0% with monotherapy, and the combination also improved OS. These findings establish axitinib plus nivolumab as an effective therapeutic strategy for tRCC and crucially demonstrate that nivolumab monotherapy has minimal activity in this rare disease, guiding future treatment decisions and trial design.

#### 3.4.3 Prostate cancer

##### 3.4.3.1 Localized high-risk disease

The management of high-risk localized prostate cancer focuses on maximizing cure while minimizing unnecessary morbidity. The ENZARAD trial evaluated systemic intensification in this setting, comparing the addition of enzalutamide to standard androgen deprivation therapy (ADT) and radiotherapy versus placebo (24). While results showed a non-significant trend toward improved metastasis-free survival (HR 0.88; 2p=0.34), they highlight that the benefit of next-generation antiandrogens is not universal in non-metastatic disease. This underscores a critical need for better patient selection tools.

The phase III trial of CAN-2409, an in situ viral immunotherapy, demonstrated that adding it to standard radiotherapy significantly reduced the risk of recurrence or death by 30% (p=0.0155) in intermediate- or high-risk patients (25). This divergence in results, between a systemic agent with modest benefit and a local immunostimulant with significant impact, emphasizes the heterogeneous nature of localized disease and the imperative for robust biomarkers to guide personalized treatment decisions, thereby avoiding overtreatment.

AI’s role extends beyond initial diagnosis into predicting therapeutic benefit. The STAMPEDE multimodal AI (MMAI) model (ArteraAI Prostate Test) integrates digital histopathology with clinical data to predict which patients with high-risk non-metastatic disease derive meaningful benefit from treatment intensification with ARPIs. In a retrospective analysis, it identified a significant biomarker-treatment interaction, showing that only patients in the highest MMAI score quartile derived a substantial survival benefit from adding ARPIs (26). A similar Duke Cancer Institute collaboration with ArteraAI developed an AI model that could identify the one-third of high-risk patients who derived no added benefit from extended (2-year) hormone therapy, potentially sparing them significant toxicity. These tools exemplify AI’s potential to enable precision medicine by guiding intensive therapy to those most likely to benefit.

##### 3.4.3.2 High-risk biochemical recurrence and oligometastatic disease

The therapeutic landscape for high-risk biochemical recurrence (BCR), defined as PSA doubling time of ≤9 months, has been fundamentally reshaped. The EMBARK trial established a new paradigm, with its final analysis confirming that adding enzalutamide to leuprolide significantly improved both metastasis-free and OS compared to leuprolide alone (8-year OS: 78.9% vs. 69.5%; HR 0.597) (27). This foundational evidence was refined by the phase III PRESTO trial, which evaluated a time-limited approach. Adding apalutamide (APA) to ADT for a finite 52-week course significantly delayed metastatic progression (MFS HR 0.80) without compromising quality of life, solidifying this as a standard option (28).

For disease progressing to a low-volume metastatic (oligometastatic) state detected by PSMA-PET, strategies aim for eradication. Early results from the phase II Metacure trial support the feasibility of combining intensified hormonal blockade with metastasis-directed stereotactic body radiotherapy (SBRT) (29).

**Figure 7:**
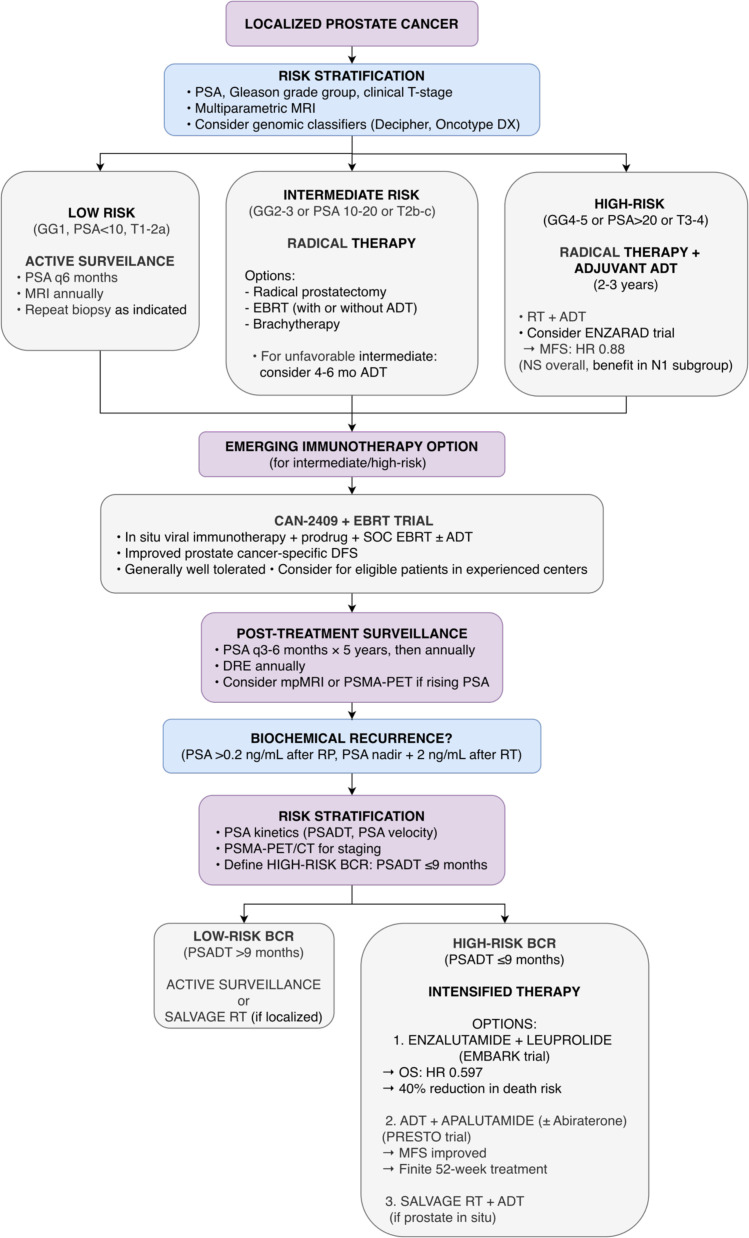
Proposed treatment algorithm for localized and biochemically recurrent prostate cancer based on 2025 ASCO/ESMO data. For localized high-risk disease, ENZARAD (Phase III) showed no overall MFS benefit but possible benefit in N1 subgroup. CAN-2409 immunotherapy (Phase III) improved prostate cancer-specific DFS. The major paradigm shift is in high-risk biochemical recurrence (PSADT ≤9 months): EMBARK (enzalutamide plus leuprolide, OS HR 0.597) and PRESTO (finite-duration apalutamide, improved MFS) establish BCR as a therapeutic intervention point. Caution: ENZARAD negative overall; EMBARK and PRESTO redefine practice but optimal regimen requires further study.

Together, these studies confirm that treatment intensification with next-generation androgen receptor pathway inhibitors fundamentally improves outcomes in high-risk BCR. They provide clinicians with a graduated toolkit, from continuous or time-limited systemic therapy to combined modality approaches, enabling treatment to be precisely tailored to an individual’s disease burden and risk profile, ultimately delaying metastatic progression and prolonging survival. The new paradigm for high-risk biochemical recurrence, established by EMBARK and PRESTO, is integrated into the proposed algorithm in **Figure 7**.

##### 3.4.3.3 Metastatic hormone-sensitive prostate cancer

In metastatic hormone-sensitive prostate cancer (mHSPC), treatment intensification with combination therapies is standard, but refining patient selection remains a priority. The 5-year follow-up from the ARCHES trial solidified the long-term OS benefit of adding enzalutamide to ADT (30).

Biomarker-driven therapy is also advancing in mHSPC. The AMPLITUDE trial established niraparib plus abiraterone as the first biomarker-directed combination in mHSPC for patients with homologous recombination repair (HRR) gene alterations, showing significantly improved radiographic PFS (rPFS) (31). Similarly, the phase III CAPItello-281 trial prospectively validated PTEN deficiency as a predictive biomarker in mHSPC. It demonstrated that adding the AKT inhibitor capivasertib to abiraterone significantly improved radiographic PFS specifically in the PTEN-deficient subgroup. This pivotal result establishes a new biomarker-directed treatment paradigm, supporting the use of PTEN testing to guide therapy selection in mHSPC (32).

Refining the role of local therapy was addressed by the STOPCAP individual participant data meta-analysis. This analysis demonstrated that prostate radiotherapy provides no OS benefit for the overall synchronous mHSPC population. However, it identified a significant interaction based on disease volume: prostate radiotherapy conferred a 21% reduction in the risk of death (HR 0.79) specifically in patients with low-volume disease, providing refined evidence for its selective use (33).

The ARASAFE trial addressed treatment intensification in mHSPC, establishing that a 2-weekly docetaxel schedule (50 mg/m²) with darolutamide and ADT offered a favorable safety profile, including reduced febrile neutropenia, compared to the standard 3-weekly schedule, without compromising efficacy (34). Concurrently, the ARANOTE trial reported that health-related quality of life (HRQoL) was preserved with darolutamide treatment, underscoring the importance of evaluating both efficacy and patient-reported outcomes (35).

**Figure 8:**
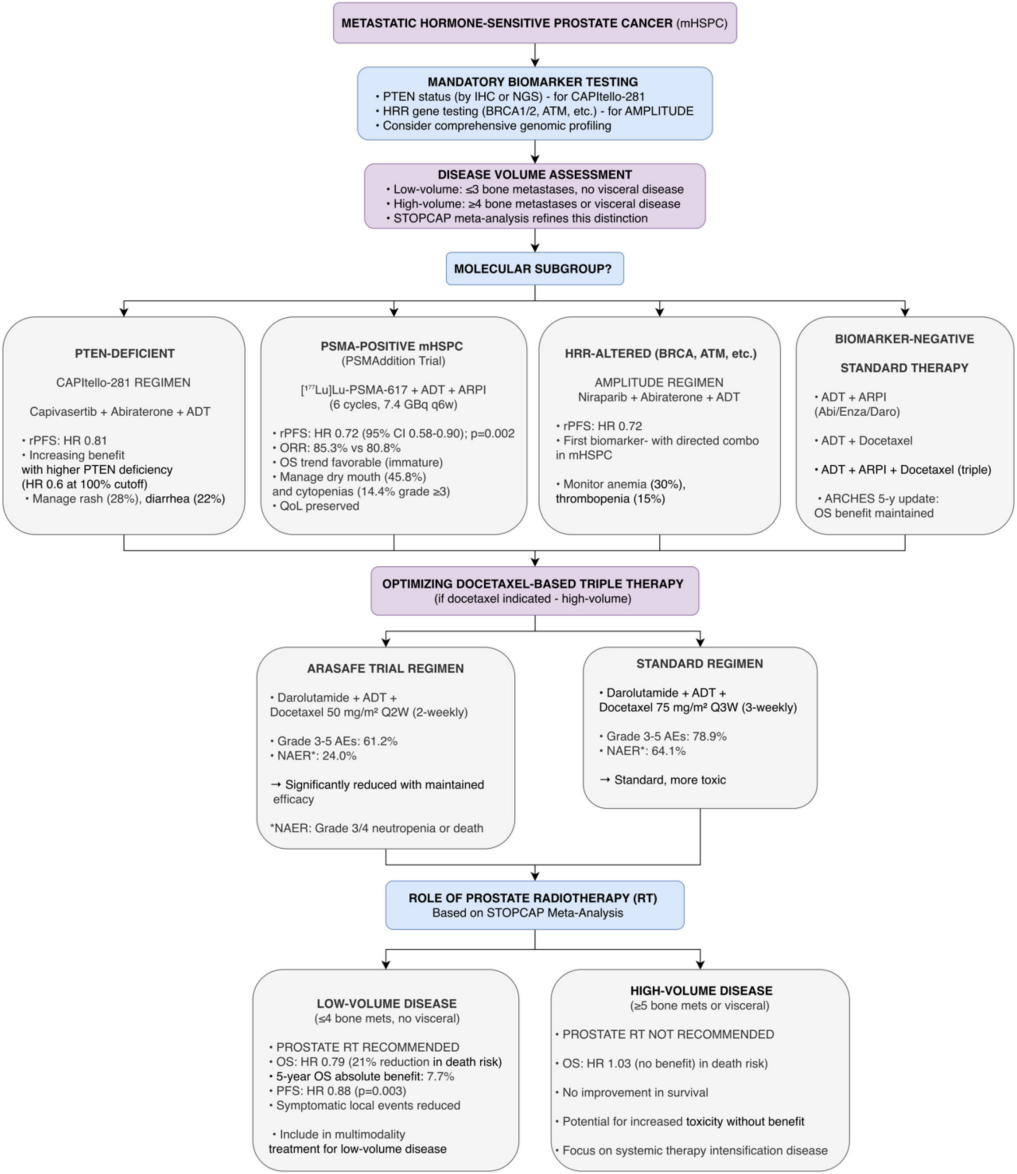
Proposed treatment algorithm for metastatic hormone-sensitive prostate cancer (mHSPC). This biomarker-driven algorithm mandates PTEN and HRR testing. For PTEN-deficient patients, capivasertib plus abiraterone is proposed (CAPItello-281 Phase III). For HRR-altered patients, niraparib plus abiraterone is proposed (AMPLITUDE Phase III). Docetaxel optimization from ARASAFE (Phase III) offers reduced-toxicity 2-weekly scheduling. Prostate radiotherapy is refined by STOPCAP meta-analysis: recommended only for low-volume disease (≤4 bone mets, no visceral; OS HR 0.79). Caution: CAPItello-281 and AMPLITUDE OS data immature; ARASAFE efficacy outcomes pending; STOPCAP awaits HORRAD inclusion. Note: Although supported by Phase III data, the integration of PTEN and HRR testing into routine practice for these combinations awaits regulatory approvals and mature overall survival confirmation.

The PSMAddition trial represents the first Phase III evaluation of radioligand therapy in the mHSPC setting. This global study randomized 1,144 patients with PSMA-positive mHSPC (as determined by [^68^Ga]Ga-PSMA-11 PET/CT) to receive [177Lu]Lu-PSMA-617 added to standard ADT plus ARPI versus ADT plus ARPI alone. At the second interim analysis (median follow-up 23.6 months), the trial met its primary endpoint with a significant improvement in rPFS favoring the [177Lu]Lu-PSMA-617 arm (HR 0.72; 95% CI 0.58-0.90; p=0.002). Overall survival showed a positive trend but was immature at the time of analysis. Safety findings were consistent with the known profile of [^177^Lu]Lu-PSMA-617: dry mouth (grade 1-2) occurred in 45.8% of patients, and grade ≥3 cytopenias were more frequent with added radioligand therapy (14.4% vs. 5.0%). Importantly, QoL was not adversely affected. This practice-changing trial establishes [177Lu]Lu-PSMA-617 as a new treatment intensification option for patients with PSMA+ mHSPC, moving radioligand therapy earlier in the disease course (36). Biomarker-directed therapy, docetaxel optimization, and refined local therapy selection in mHSPC are summarized in **Figure 8**.

##### 3.4.3.4 Metastatic castration-resistant prostate cancer

The metastatic castration-resistant prostate cancer (mCRPC) treatment landscape is characterized by sequential use of novel hormonal agents, chemotherapy, radiopharmaceuticals, and targeted therapies. The TALAPRO-2 trial reinforced the efficacy of combining the PARP inhibitor talazoparib with enzalutamide in patients with HRR-deficient mCRPC, with profound benefit observed in those with alterations in the BRCA1/2 and PALB2 genes (37). The search for more tolerable combinations continues, with the phase Ib/II PETRANHA trial introducing saruparib, a PARP1-selective inhibitor, which combined with an ARPI showed promising activity and a favorable hematological safety profile in ARPI-naive patients (38).

**Figure 9:**
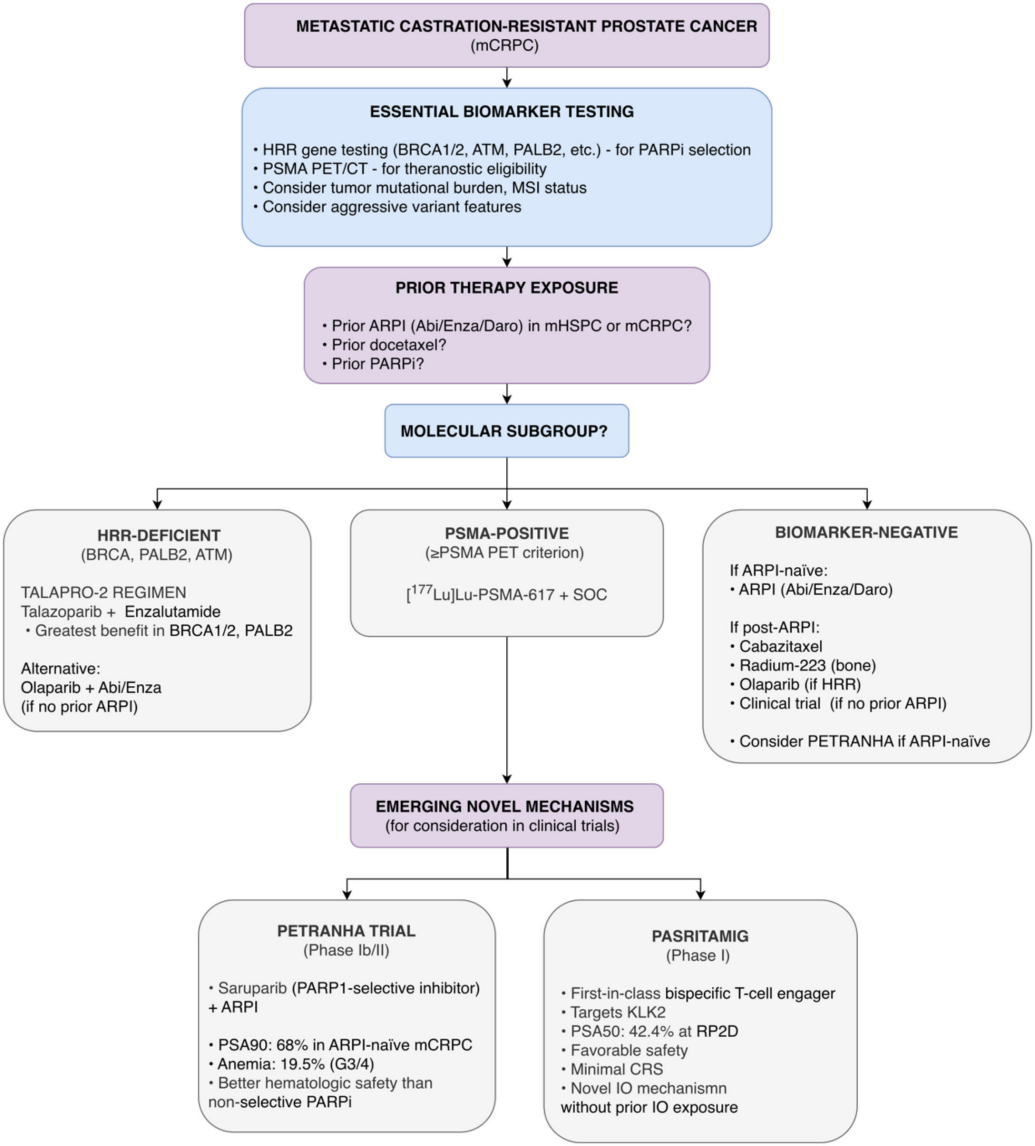
Proposed treatment algorithm for metastatic castration-resistant prostate cancer (mCRPC) based on 2025 ASCO/ESMO data. This algorithm integrates biomarker-directed therapy (HRR status, PSMA PET/CT). For HRR-deficient patients, talazoparib plus enzalutamide is shown based on TALAPRO-2 exploratory analyses. Note: In alignment with ESMO 2026 guidelines, PARPi-based combinations (e.g., talazoparib plus enzalutamide) are primarily considered for patients with BRCA alterations who have not received prior ARPI therapy; for those with prior ARPI exposure, PARPi monotherapy (olaparib or rucaparib) is preferred. For PSMA-positive patients, [^177^Lu]Lu-PSMA-617 plus SOC is included. Emerging novel mechanisms from early-phase trials are shown: saruparib (PARP1-selective inhibitor, PETRANHA Phase Ib/II), and pasritamig (KLK2xCD3 T-cell engager, Phase I). Caution: TALAPRO-2 analyses are exploratory; PETRANHA, and pasritamig are early-phase requiring confirmation.

Novel immune and targeted strategies are emerging. Pasritamig, a first-in-class bispecific T-cell engager targeting prostate-specific KLK2, showed promising early anti-tumor activity and a manageable safety profile in heavily pre-treated mCRPC patients in a Phase I study, with 42.4% of patients achieving a ≥50% PSA reduction at the recommended dose (39). **Figure 9** proposes a treatment framework for mCRPC integrating novel mechanisms and sequencing principles.

#### 3.4.4 Testicular cancer

The management of stage IIA/B seminoma has been redefined by the long-term results of the SAKK 01/10 trial, which validated a de-escalated, organ-preserving approach. This multicenter, single-arm phase II study evaluated a regimen of single-dose carboplatin (AUC 7) followed by involved-node radiotherapy (30 Gy for IIA, 36 Gy for IIB). With a median follow-up of 8 years, the trial demonstrated excellent and durable efficacy: the 10-year PFS rate was 92.8% (95.2% for IIA, 91.3% for IIB), with no disease progression events recorded since the primary analysis. Overall survival at 10 years was 99.1%. Critically, the regimen showed minimal long-term toxicity, with only one late thromboembolic event possibly related to treatment and no increase in treatment-related secondary malignancies compared to historical standards. These results firmly establish the combination of single-dose carboplatin and involved-node radiotherapy as a new standard of care for stage IIA/B seminoma, offering high cure rates while minimizing both acute and late treatment-related morbidity (40).

#### 3.4.5 Penile cancer

The randomized trial of adjuvant chemotherapy in high-risk penile carcinoma provided much-needed evidence in this rare malignancy. This phase III, randomized, non-inferiority study compared adjuvant platinum plus paclitaxel versus platinum plus 5-fluorouracil in patients with high-risk penile cancer after resection (41). The primary endpoint was OS, which showed non-inferiority with the paclitaxel-containing regimen (HR 1.32). The safety profile showed expected toxicities, including more hematologic toxicities (31.8% vs. 13% grade ≥3) and gastrointestinal toxicities (31.8% vs. 4.3% grade ≥3). This establishes a new adjuvant standard for this rare cancer.

### 3.5 Summary of evidence tables

**Table 1** provides a comprehensive overview of all 40 included studies, summarizing key characteristics including tumor type, disease setting, study design, interventions, primary endpoints, and safety findings. This table serves as a quick reference for clinicians and researchers seeking to understand the breadth of evidence presented in 2025.

## 4 Discussion

The year 2025 has delivered transformative advances across the spectrum of genitourinary malignancies, reshaping therapeutic paradigms and establishing new standards of care. This systematic review synthesizes evidence from 40 prospective phase II and III trials presented at major oncology conferences and published in high-impact journals, revealing several interconnected themes that are collectively redefining GU oncology practice.

### 4.1 Implications for clinical practice by tumor type

Bladder cancer: The 2025 data have fundamentally reshaped treatment algorithms across disease stages. For non-metastatic disease, NIAGARA (4) establishes perioperative durvalumab as a new standard for cisplatin-eligible MIBC, while KEYNOTE-905/EV-303 (5) provides a transformative option for cisplatin-ineligible patients with enfortumab vedotin plus pembrolizumab. These advances move effective systemic therapy earlier in the disease course, with the goal of improving cure rates. In the metastatic setting, EV-302/KEYNOTE-A39 (9) establishes enfortumab vedotin plus pembrolizumab as the new frontline standard, offering unprecedented survival benefits. The RC48-C016 (11, 12) trial creates a biomarker-defined frontline option for HER2-positive patients, emphasizing the importance of HER2 testing in mUC. These developments create a hierarchical approach to frontline therapy: enfortumab vedotin plus pembrolizumab for all comers, with disitamab vedotin plus toripalimab as a preferred option for HER2-positive patients. The divergent results in NMIBC (POTOMAC positive (1), ALBAN negative (2)) highlight the complexity of immunotherapy integration in this setting and suggest potential differences between PD-1 and PD-L1 inhibitors that warrant further investigation.

Kidney cancer: The long-term follow-up of CheckMate 214 (17) confirms the durability of benefit with nivolumab plus ipilimumab in intermediate/poor-risk metastatic clear cell RCC, solidifying its position as a standard first-line option. The OPTIC RCC (18) trial represents a conceptual advance toward transcriptomic phenotype-guided therapy, moving beyond clinical risk stratification alone. While not yet ready for routine clinical implementation, this approach demonstrates the potential of molecular profiling to optimize treatment selection. In the adjuvant setting, RAMPART (15) expands options beyond pembrolizumab, providing clinicians with choices based on individual patient risk tolerance and preference. For rare subtypes, AREN1721 (23) establishes axitinib plus nivolumab as a new standard for translocation RCC, addressing a significant unmet need.

Prostate cancer: The EMBARK (27) and PRESTO (28) trials represent a paradigm shift in the management of high-risk biochemical recurrence, demonstrating for the first time that systemic therapy can improve OS in this setting. This fundamentally redefines biochemical recurrence as a therapeutic state warranting life-prolonging intervention. In metastatic hormone-sensitive disease, biomarker-directed approaches are advancing. The CAPItello-281 trial establishes PTEN deficiency as a predictive biomarker for AKT inhibitor combination therapy (32), complementing other biomarkers such as HRR alterations that guide the use of PARP inhibitors in this setting (31). The ARASAFE (34) trial provides practical guidance on toxicity management through optimized docetaxel scheduling. The STAMPEDE MMAI model (26) introduces artificial intelligence into clinical decision-making, offering the potential to personalize treatment intensity based on predicted benefit. The PSMAddition trial introduces radioligand therapy as a new treatment intensification option for PSMA-positive mHSPC, demonstrating improved rPFS when added to standard ADT plus ARPI. This moves [^177^Lu]Lu-PSMA-617 from the mCRPC setting into earlier disease stages, expanding the therapeutic arsenal and reinforcing the importance of PSMA PET/CT as a biomarker for treatment selection (36). These developments underscore the critical importance of comprehensive molecular testing and treatment optimization.

Penile cancer: The establishment of adjuvant platinum plus paclitaxel as superior to platinum plus 5-fluorouracil (41) provides much-needed evidence-based guidance in this rare malignancy, where clinical trials have historically been scarce. This advance represents an important step forward in standardizing care for this challenging disease.

### 4.2 Limitations of the presented evidence

Several important limitations must be acknowledged when interpreting the 2025 data. First, a significant portion of the evidence (31 of 40 studies, 77.5%) comes from conference presentations with immature follow-up [e.g., 3, 4, 5, 6, 7, 8, 10, 11, 13, 15–20, 22–25, 27–31, 33–38, 40, 41]. Final publications with extended follow-up may refine effect sizes, particularly for OS endpoints. Second, the high cost of novel therapies and companion diagnostics poses a formidable barrier to equitable global access. Third, as therapeutic options multiply, determining the optimal sequence of therapy becomes increasingly complex, for instance, how to best sequence ADCs such as enfortumab vedotin (5, 9) and disitamab vedotin (11, 12) with immunotherapy (4, 10) in urothelial carcinoma, or how to sequence novel hormonal agents (27, 28, 30, 32, 34, 35), PARP inhibitors (31, 37, 38), and radiopharmaceuticals (36) in prostate cancer. Fourth, the generalizability of trial findings to real-world populations, which are often older and have more comorbidities than trial participants, requires further study. Finally, the increasing complexity of treatment combinations raises challenges in toxicity management and requires specialized expertise that may not be available in all clinical settings.

### 4.3 Common themes across genitourinary malignancies

Across the spectrum of GU cancers, several unifying themes emerge from the 2025 data. First, biomarker-driven therapy has made significant strides towards clinical reality. This is exemplified by HER2 in urothelial carcinoma (RC48-C016) and HRR alterations in prostate cancer (AMPLITUDE), while promising signals for PTEN (CAPItello-281) and transcriptomic profiling (OPTIC RCC) pave the way for future refinement of treatment selection. Second, ADCs have established themselves as backbone therapies, with enfortumab vedotin revolutionizing urothelial cancer and disitamab vedotin creating a new biomarker-defined category. Third, treatment optimization and de-escalation are gaining prominence, as demonstrated by shorter chemotherapy duration (DISCUS), reduced-toxicity docetaxel scheduling (ARASAFE), and refined patient selection for local therapy (STOPCAP). Fourth, immunotherapy continues to expand into earlier disease stages, with perioperative approaches in bladder cancer (NIAGARA, KEYNOTE-905) and adjuvant strategies in renal cancer (RAMPART). Finally, artificial intelligence is beginning to fulfill its promise, with the STAMPEDE MMAI model providing a powerful proof-of-concept that digital pathology combined with clinical data has the potential to personalize treatment intensity, pending prospective validation and implementation studies. Radioligand therapy is moving earlier in the disease course, with PSMAddition demonstrating efficacy in mHSPC following the established benefit in mCRPC from the VISION and PSMAfore trials. These converging trends signal a maturing field where the focus is shifting from merely adding therapies to intelligently selecting, sequencing, and optimizing them for individual patients.

### 4.4 Future directions and unresolved questions by tumor type

Bladder cancer: Key unanswered questions include optimal sequencing after progression on enfortumab vedotin plus pembrolizumab (9), validation of predictive biomarkers beyond HER2 (11, 12), and development of strategies to manage cumulative toxicities from ADCs (5, 9, 11, 12). Future research should also explore novel combinations and mechanisms to overcome resistance to current standards.

Kidney cancer: Priorities include validation of transcriptomic classifiers in larger prospective trials (18), development of effective therapies for non-clear cell histologies, and optimization of treatment sequences in the era of multiple active agents (15, 17, 21, 22). Research into novel mechanisms beyond VEGF and immune checkpoint inhibition is also needed.

Prostate cancer: Critical questions include optimal sequencing of novel agents in metastatic castration-resistant disease (37–39), validation of artificial intelligence models in diverse populations (26), development of strategies to overcome treatment resistance, and optimization of treatment duration and de-escalation approaches (27, 28, 34). Research into novel mechanisms and combinations for aggressive variant prostate cancer remains a priority.

Penile and other rare GU cancers: There is a continued need for dedicated clinical trials in rare malignancies (41), development of biomarkers to guide therapy, and strategies to improve access to effective treatments globally.

## 5 Conclusions

The year 2025 stands as a definitive watershed moment in genitourinary oncology. The convergence of high-impact data has forged multiple new standards of care across bladder, kidney, prostate, and penile cancers, fundamentally reshaping therapeutic paradigms. These developments collectively herald a new era of more effective, tailored, and patient-centered care, characterized by biomarker-driven treatment selection, integration of novel therapeutic mechanisms, and application of artificial intelligence to optimize clinical decision-making.

However, these remarkable advances also present significant challenges for the global oncology community. The rapid integration of complex, biomarker-driven strategies into diverse healthcare systems requires thoughtful implementation. The navigation of escalating treatment costs and resource constraints demands innovative solutions. And the strategic prioritization of future research must address persistent knowledge gaps, particularly in treatment sequencing and overcoming resistance.

Successfully meeting these challenges will be essential to ensure that the promise of the 2025 breakthroughs is fully realized for all patients affected by genitourinary cancers. As we move forward, a continued commitment to rigorous clinical research, equitable access to innovations, and patient-centered care will be paramount in translating these scientific advances into meaningful improvements in patient outcomes worldwide.

## Data Availability

The raw data supporting the conclusions of this article will be made available by the authors, without undue reservation.

## Glossary

ADCs: Antibody-drug conjugates
ADT: Androgen Deprivation Therapy
AE: Adverse Event
AI: Artificial Intelligence
AR: Androgen Receptor
ARPI: Androgen Receptor Pathway Inhibitor
BCG: Bacillus Calmette-Guérin
BCR: Biochemical Recurrence
BICR: Blinded Independent Central Review
CI: Confidence Interval
CR: Complete Response
DFS: Disease-Free Survival
EFS: Event-Free Survival
eGFR: Estimated Glomerular Filtration Rate
GI: Gastrointestinal
HR: Hazard Ratio
HRQoL: Health-Related Quality of Life
HRR: Homologous Recombination Repair
IO: Immuno-Oncology/Immunotherapy
MFS: Metastasis-Free Survival
MPR: Major Pathologic Response
MRI: Magnetic Resonance Imaging
NMIBC: Non-Muscle-Invasive Bladder Cancer
ORR: Objective Response Rate
OS: Overall Survival
PCa: Prostate Cancer
pCR: Pathologic Complete Response
PFS: Progression-Free Survival
PR: Partial Response
PSA: Prostate-Specific Antigen
PSMA: Prostate-Specific Membrane Antigen
QoL: Quality of Life
RCC: Renal Cell Carcinoma
RCT: Randomized Controlled Trial
rPFS: Radiographic Progression-Free Survival
RT: Radiotherapy
SBRT: Stereotactic Body Radiotherapy
SOC: Standard of Care
TEAE: Treatment-Emergent Adverse Event
TKI: Tyrosine Kinase Inhibitor
TRAE: Treatment-Related Adverse Event
TURBT: Transurethral Resection of Bladder Tumor
UTUC: Upper Tract Urothelial Carcinoma
VEGF: Vascular Endothelial Growth Factor
